# Third-generation ultralong-term latent heat storage glass based on a sugar alcohol mixture

**DOI:** 10.1126/sciadv.aei4311

**Published:** 2026-09-23

**Authors:** Kaixin Dong, Akihito Sagara, Qingda Li, Kepeng Huang, Yuto Shimizu, Tomokazu Nakamura, Joshua Chidiebere Mba, Keita Tanahashi, Melbert Jeem, Zhonghao Rao, Takahiro Nomura

**Affiliations:** ^1^ Faculty of Engineering, Hokkaido University, Kita 13 Nishi 8, Kita-ku, Sapporo 060-8628, Japan.; ^2^ Graduate School of Engineering, Hokkaido University, Kita 13 Nishi 8, Kita-ku, Sapporo 060-8628, Japan.; ^3^ College of Mechanical and Electronic Engineering, Northwest A&F University, 22 Xinong Road, Yangling 712100, Shaanxi, China.; ^4^ Institute of Multidisciplinary Research for Advanced Materials, Tohoku University, 2-1-1, Katahira, Aoba-ku, Sendai, Miyagi 980-8577, Japan.; ^5^ Hebei Engineering Research Center of Advanced Energy Storage Technology and Equipment, School of Energy and Environmental Engineering, Hebei University of Technology, Tianjin, 300401, China.; ^6^ Hebei Key Laboratory of Thermal Science and Energy Clean Utilization, School of Energy and Environmental Engineering, Hebei University of Technology, Tianjin, 300401, China.

## Abstract

Long-term thermal storage can decarbonize heat utilization by resolving mismatches between heat supply and demand. However, common heat storage materials cannot store energy for long periods. Here, we report an ultralong-term latent heat storage “glass” based on a ternary eutectic sugar alcohol mixture. The as-prepared material exhibits a solid–liquid phase change and glass transition at 423 and 289 K, respectively, with heat release via cold crystallization at 346 K. Remarkably, the glass transition temperature increases to 313 K after aging at ambient temperature for 12 hours due to structural relaxation. This phase change material (PCM) conserves the latent heat of fusion at 423 K for at least 180 days in the glass state at ambient temperature. These results establish a third-generation latent heat storage mode, retaining the charged state as a kinetically arrested amorphous solid and thereby fundamentally shifting the stability criterion from supercooled-liquid nucleation suppression to glass-transition and structural-relaxation kinetics.

## INTRODUCTION

The global shift in focus away from fossil fuels necessitates decarbonized heat utilization systems. According to the International Energy Agency, heat accounted for almost half of the total final energy consumption in 2022 (*1*). Heat from solar energy is abundant, and every energy conversion ultimately generates heat. However, a large amount of this energy is unrecovered and dispersed into the environment because of the spatiotemporal mismatch between heat sources and demands (*2*). Using the excess solar heat in summer for winter or efficiently storing, transporting, and utilizing waste heat from industrial processes would significantly promote the realization of a decarbonized society.

**Fig. 1.**
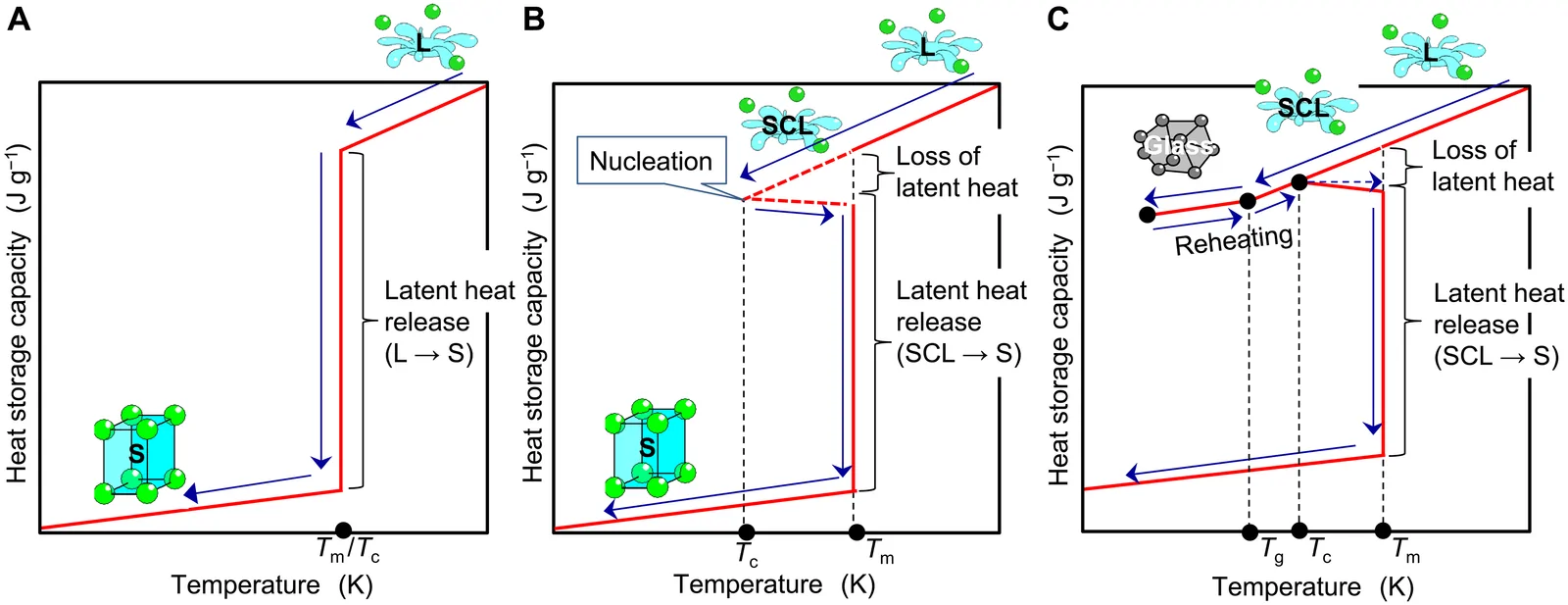
Principles of latent heat storage. (**A**) Liquid type (first generation), (**B**) supercooled type (second generation), and (**C**) glass type (third generation) latent heat storage capacity as a function of temperature. *T*_m_, melting temperature; *T*_c_, crystallization temperature; *T*_g_, glass transition temperature; L, liquid; SCL, supercooled liquid; S, solid.

Latent heat storage phase change materials (PCMs) have been studied to overcome the spatiotemporal mismatch in heat utilization. There are first and second-generation technologies using solid-liquid phase transition of PCMs. Figure 1A shows the working principle of a typical PCM, referred to as first-generation latent heat storage in this study. Such a PCM absorbs heat as the latent heat of fusion at the melting temperature (*T*_m_) and stores the heat in the liquid state at temperatures beyond *T*_m_. Last, the stored heat is released during solidification at *T*_m_ (= Crystalization temperature, *T*_c_). Such a PCM exhibits a high heat storage density owing to the use of latent heat and a constant-temperature heat supply at *T*_m_. Therefore, the first-generation latent heat storage has been investigated for thermal energy storage (TES) purposes lasting from several hours to several days such as industrial waste heat recovery and transportation systems (*3*–*8*), thermal energy storage for concentrated solar power plant (*9*, *10*), and Carnot batteries (*11*, *12*). However, heat storage requires PCM to be maintained at *T*_m_, which typically exceeds the ambient temperature. Significant unavoidable heat loss poses a crucial challenge in the long-term storage of 1st-generation PCMs.

Figure 1B shows the principle of a supercooled liquid (SCL) PCM, referred to as 2nd generation latent heat storage in this study. The SCL-PCM stores the heat as latent heat in a SCL state at temperatures below the *T*_m_ of the PCM (∼298 K). The stored heat is released by initiating the crystallization of the SCL and inducing nucleation via mechanical vibration or agitation (*13*), cold crystallization (*14*), the use of nucleation agents (*15*, *16*), bubbling (*17*), or electrical pulses (*18*). Hydrate salt (*19*–*22*) or sugar alcohol has been studied as SCL-PCMs because they can maintain their SCL states at temperatures close to ambient temperature. Sugar alcohols have been widely studied owing to their low costs, high heat storage density, and good thermal stability. These alcohols include xylitol (*23*, *24*), threitol (*25*), erythritol (*17*, *26*–*30*), mannitol (*31*), and their mixtures (*32*). The SCL stability is key to the long-term heat storage of SCL-PCMs. Various additives have been introduced into PCMs to increase the stabilities of these SCLs. Yazdani *et al.* (*33*) successfully developed a supercooled erythritol. Ionic cross-linked matrices of polyvinyl alcohol and sodium citrate were introduced into the erythritol PCM, which enabled the material to maintain a SCL state instead of undergoing crystallization during cooling to an extremely low temperature of 253 K. The SCL could then be reheated to release heat via cold crystallization at approximately 293 K. Li *et al.* (*34*) developed a SCL-type erythritol PCM stabilized with NaOH. Hydrogen bonding between the PCM and NaOH increased the nucleation energy barrier of the PCM, hindering its orderly arrangement during cooling. Yang *et al.* (*30*) incorporated carrageenan into an erythritol PCM and increased the solid-liquid interfacial energy, effectively stabilizing the PCM down to 243 K. SCL-PCMs should enable long-term or seasonal TES, but SCLs are prone to unavoidable unexpected crystallization due to mechanical shocks, vibration, and heterogeneous nucleation caused by impurities and the walls of heat storage tanks. In addition, additives for use in stabilizing supercooled states decrease the heat storage capacity and increase the overall material cost.

Here, we define third-generation latent-heat storage as an operational materials class rather than simply a chronological extension from liquid to supercooled liquid to glass. In first-generation PCMs, the charged state is a liquid above *T*_m_ and heat is released during solidification. In second-generation supercooled-liquid PCMs, the charged state is a metastable liquid below *T*_m_ and heat retention depends on suppressing nucleation and crystal growth. In the proposed third-generation glass PCM (G-PCM), cooling brings the system through *T*_g_ into a kinetically arrested amorphous solid, and heat is later released by cold crystallization on reheating. This classification concerns the physical state and heat-release pathway; it does not imply unlimited storage stability, which remains governed by the coupled kinetics of structural relaxation and crystallization. These distinctions are summarized in table S1.

In this study, the glassy state is treated as a kinetically arrested, nonequilibrium amorphous solid. When a liquid is cooled sufficiently rapidly to avoid crystallization, molecular mobility decreases and the material falls out of equilibrium near its calorimetric *T*_g_ (*35*). Calorimetric *T*_g_ is an operational kinetic quantity that depends on scan rate, thermal history, and structural state; it is not a thermodynamic phase boundary. The glass therefore remains metastable and can undergo structural/enthalpy relaxation or devitrification over time, particularly near or above its initial *T*_g_. On reheating, mobility increases and, if crystallization precedes melting, the stored enthalpy is released as the cold-crystallization enthalpy *L*_c_. Accordingly, practical stability must be specified by temperature, duration, sample geometry, and environment rather than inferred from *T*_g_ alone.

We therefore propose a G-PCM as a third-generation latent heat storage concept (Fig. 1C). The material is charged by melting near 423 K, cooled into an amorphous solid, and subsequently releases heat through cold crystallization near 346 K. We selected a ternary eutectic mixture of mannitol, dulcitol, and inositol because compositional complexity and hydrogen-bonding interactions can impede crystallization while preserving a high fusion enthalpy (*36*, *37*). The present measurements demonstrate vitrification without rapid quenching, heat release by cold crystallization, measurable capacity after 180 days at 295 ± 1 K, and reproducibility over repeated laboratory thermal cycles. The total fusion enthalpy *L*_m_ and the retrievable cold crystallization enthalpy *L*_c_ are distinguished below, and storage limits are empirically defined.

## RESULTS AND DISCUSSION

**Fig. 2.**
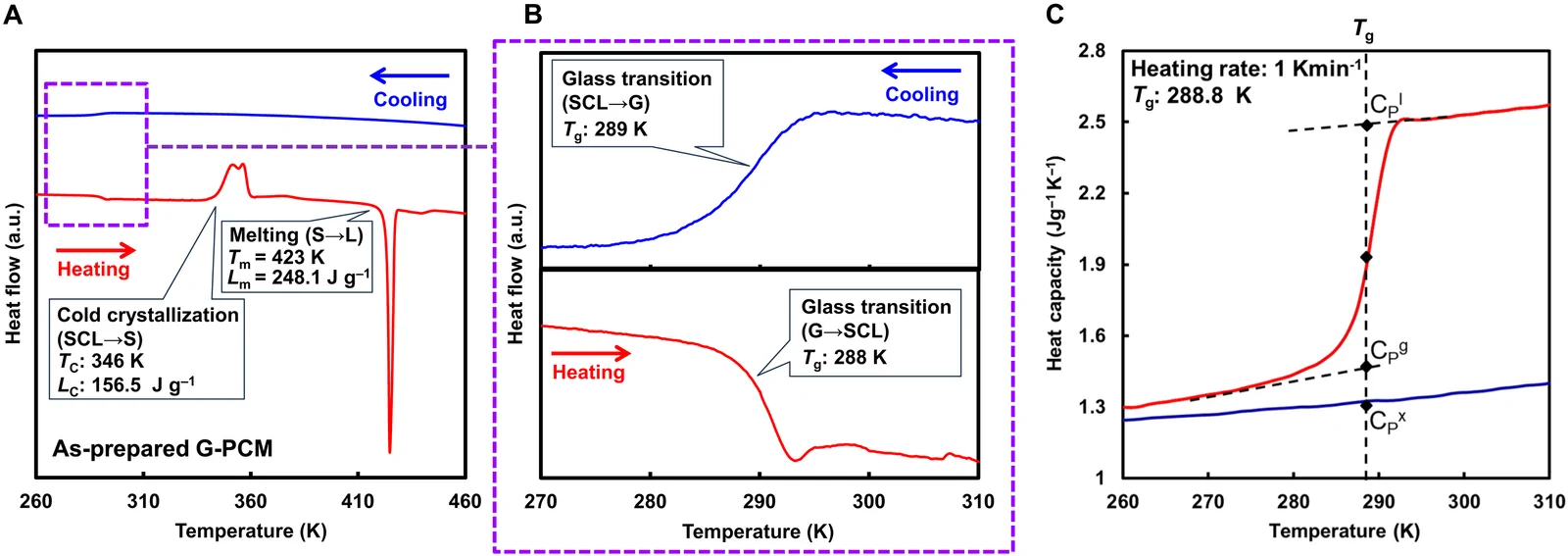
Glass transition characteristics of amorphous PCM. (**A** and **B**) DSC thermograms of the G-PCM in one thermal cycle (*L*_m_, latent heat of melting; *L*_c_, latent heat of crystallization) and (**C**) specific heats of the glass (red) and crystalline (blue) states of the G-PCM. G, glass.

An initial calorimetric *T*_g_ near ambient temperature was used as a screening criterion, not as a guarantee of long-term stability. The solid-liquid phase change and glass transition behavior of the ternary eutectic mixture of mannitol, dulcitol, and inositol was evaluated by differential scanning calorimetry (DSC). Figure 2 (A and B) shows thermograms acquired at 2 K min^−1^. During cooling from 453 K, no exothermic crystallization peak was observed; a heat-capacity step occurred near 289 K. During subsequent heating from 260 K, a corresponding glass transition step appeared near 288 K, followed by an exothermic cold crystallization peak at 346 K and an endothermic melting peak at 423.5 K. These temperatures are scan rate– and history-dependent operational values.

Figure 2C shows the heat capacities at constant pressure (*C*_p_) of the glass and crystalline states of the G-PCM as functions of temperature. Expectedly, the heat capacity of the crystalline sample increases almost linearly with increasing temperature, whereas that of the amorphous sample increases sharply at ∼*T*_g_. These results confirm the glass transition of the G-PCM (at 288 K) and demonstrate its heat storage capacity in the glassy (amorphous solid) state. Mixing three sugar alcohols with different structures considerably increases the intermolecular interactions, particularly hydrogen bonding, which is realized using additives in previous studies by Nakanishi *et al.* (*38*). The strong intermolecular interactions restrict molecular motion, resulting in a high *T*_g_ of approximately room temperature and a high heat release temperature. Numerous researchers have reported the roles of hydrogen bonding in the glass transition and supercooling of organic materials (*30*, *33*, *34*).

Scan rate sensitivity was evaluated using conventional DSC and Flash-DSC (figs. S3 to S5). No exothermic event attributable to crystallization was detected during cooling at 1 to 50 K min^−1^ in conventional DSC or at 0.1 to 10 K s^−1^ (6 to 600 K min^−1^) in Flash-DSC. Because crystallization was absent even at the lowest tested rate, the exact critical cooling rate (*R*_c_) was not reached; the conventional DSC result establishes an operational upper bound of *R*_c_ ≤ 1 K min^−1^ for the milligram-scale specimen under the stated conditions and detection sensitivity. The subsequent heating scans retained the glass transition–cold crystallization–melting sequence. Cooling history principally affected the apparent heating-branch *T*_g,onset_, whereas *T*_c,onset_ and *T*_m,peak_ remained nearly constant under a common heating rate. Increasing the heating rate shifted the apparent *T*_g,onset_ and resolved *T*_c,onset_ to higher temperatures, reflecting the kinetic competition among structural relaxation, nucleation, and crystal growth (*39*–*44*). These small-sample results show that rapid quenching is unnecessary for vitrification but should not be extrapolated directly to bulk systems, for which thermal gradients and heterogeneous nucleation require independent validation (*14*). On the other hand, no systematic deterioration after 100 DSC cycles between 243 and 453 K (fig. S1) under the investigated melting–vitrification–cold crystallization protocol. The vitrification point and cold crystallization temperature remain stable, and the degradation of the latent heat is extremely low, indicating the durability and stability of the G-PCM, which are essential in long-term applications. The thermal analysis of the G-PCM reveals that the ternary sugar alcohol PCM samples prepared are vitrified at temperatures below their *T*_m_ values while maintaining their heat storage states, and *L*_c_ is released via reheating, i.e., cold crystallization. Therefore, the heat storage state is maintained in an amorphous state at below *T*_g_, and *L*_c_ is released only by increasing the temperature.

Yazdani *et al.* (*33*) reported a sugar-alcohol PCM with glass transition commencing below 243 K. At temperatures near ambient, such a material can remain a supercooled liquid whose stability is sensitive to nucleation and whose heat-release temperature is comparatively low. In the present G-PCM, vitrification produces an amorphous solid near ambient temperature and reduces sensitivity to mechanically triggered flow or handling. Nevertheless, a glass is still metastable: vibration resistance does not eliminate thermally activated nucleation, surface effects, or long-term devitrification. The relevant comparison with supercooled-liquid PCMs is therefore the charged state and release pathway, together with experimentally established temperature–time stability, rather than an assertion of absolute immunity to crystallization.

The structural-relaxation timescale of the as-prepared G-PCM was estimated using the Adam-Gibbs-Vogel relation and the heating rate dependence of the apparent calorimetric *T*_g_ (fig. S2) (*45*–*48*). The slope of ln(*q*) versus 1000/T_g_ gives an apparent activation enthalpy of 599.5 kJ mol^−1^ and a fragility index *m* of 108.5. The resulting τ(*T*) describes mean structural relaxation under the assumptions of the model; it is neither a crystallization-induction time nor a guaranteed storage lifetime. Experimental storage tests are therefore required to define duration-specific operating limits.

**Fig. 3.**
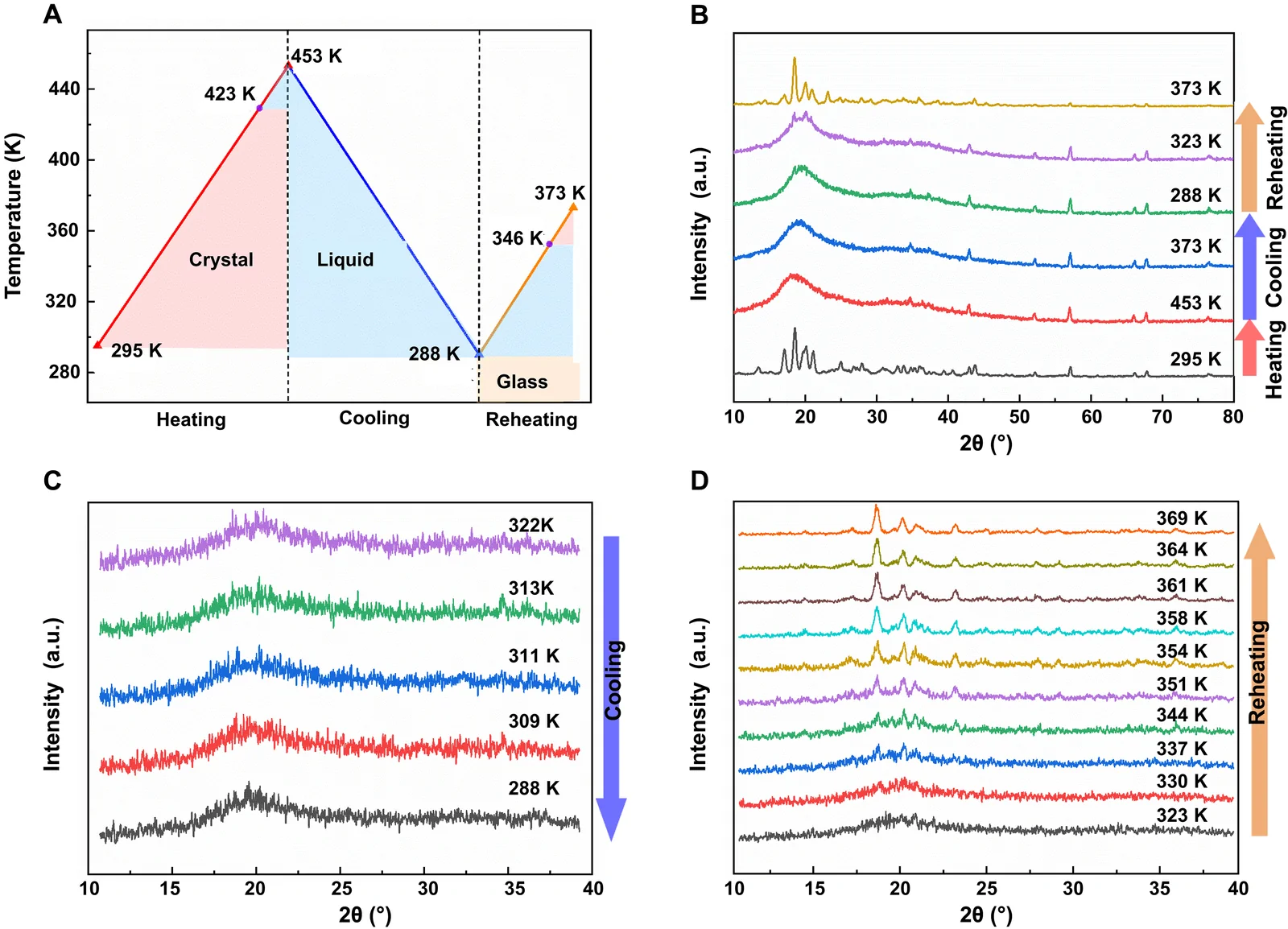
In situ XRD patterns of the G-PCM at different temperatures. (**A**) Temperature history of the G-PCM, (**B**) in situ XRD patterns of the G-PCM at different temperatures over one heating-cooling cycle, (**C**) upon cooling at approximately room temperature, and (**D**) upon reheating.

Figure 3 shows the in situ x-ray diffraction (XRD) patterns of the G-PCM during heating and cooling from different initial states. As shown in the temperature history (Fig. 3A), the as-prepared solid crystalline sample was heated from 295 to 453 K, cooled to 288 K, and lastly reheated to 373 K for cold crystallization. The as-prepared G-PCM displays a crystalline pattern representing the ternary eutectic mixture, with the peaks representing α-Al_2_O_3_ derived from the sample holder (Fig. 3B). When the sample is melted at 453 K, its XRD pattern is consistent with that of the liquid phase, and crystallization is not observed during cooling to a temperature considerably lower than *T*_m_ (373 K). The sample remains amorphous, even when cooled to 288 K, which confirms the vitrification of the G-PCM during cooling. During reheating, the G-PCM first displays an amorphous pattern, then crystalline peaks begin to manifest with increasing temperature, and last, the sample transforms into a completely crystalline state.

Figure 3 (C and D) shows rapid XRD scans during cooling and reheating, respectively. The absence of sharp diffraction peaks after cooling to 288 K is consistent with formation of an amorphous state within the measurement sensitivity. During reheating, crystalline peaks begin to emerge near 337 K and stabilize above 358 K, confirming cold crystallization. These measurements establish the phase sequence for the tested thermal cycle but do not, by themselves, prove indefinite room-temperature stability or exclude a crystalline fraction below the detection limit.

**Fig. 4.**
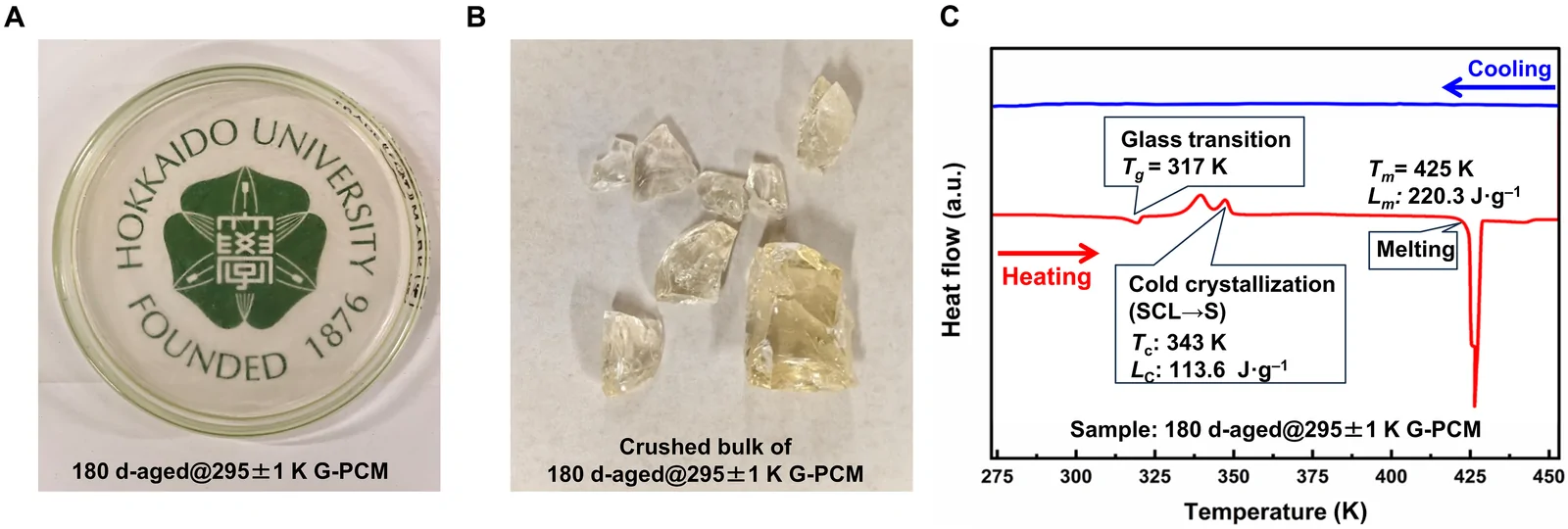
Long-term storage response of the G-PCM at 295 ± 1 K under laboratory air for 180 days. (**A**) Macroscopic appearance of the stored specimen, (**B**) crushed specimen, and (**C**) reheating DSC thermogram. The aged material retained glass transition, cold crystallization, and melting features; its apparent that reheating *T*_g_ was 317 K and *L*_c_ was 113.64 J g^−1^. Slight discoloration was observed. The exposure was not humidity controlled.

After storage at 295 ± 1 K for 180 days under laboratory air, the specimen did not develop the opaque-white appearance associated with extensive devitrification but showed a slight yellow/amber tint (Fig. 4, A and B). This optical change is distinct from the cloudy or white appearance after 30 days at 303 to 308 K, which is consistent mainly with devitrification-induced light scattering (figs. S6 and S7). Reheating DSC retained the glass transition, cold crystallization, and melting features, with *L*_c_ = 113.64 J g^−1^ (72.6% of the fresh value) and an apparent *T*_g_ of 317 K (Fig. 4C). The amber color is chemically nonspecific; trace thermally activated or oxidative products from processing in air cannot be excluded, and the data do not establish a quantitative causal relationship between yellowing and enthalpy loss. Detailed analysis of the optical changes, analytical limitations, and containment implications is provided in the Supplementary Materials (figs. S6 to S9).

**Fig. 5.**
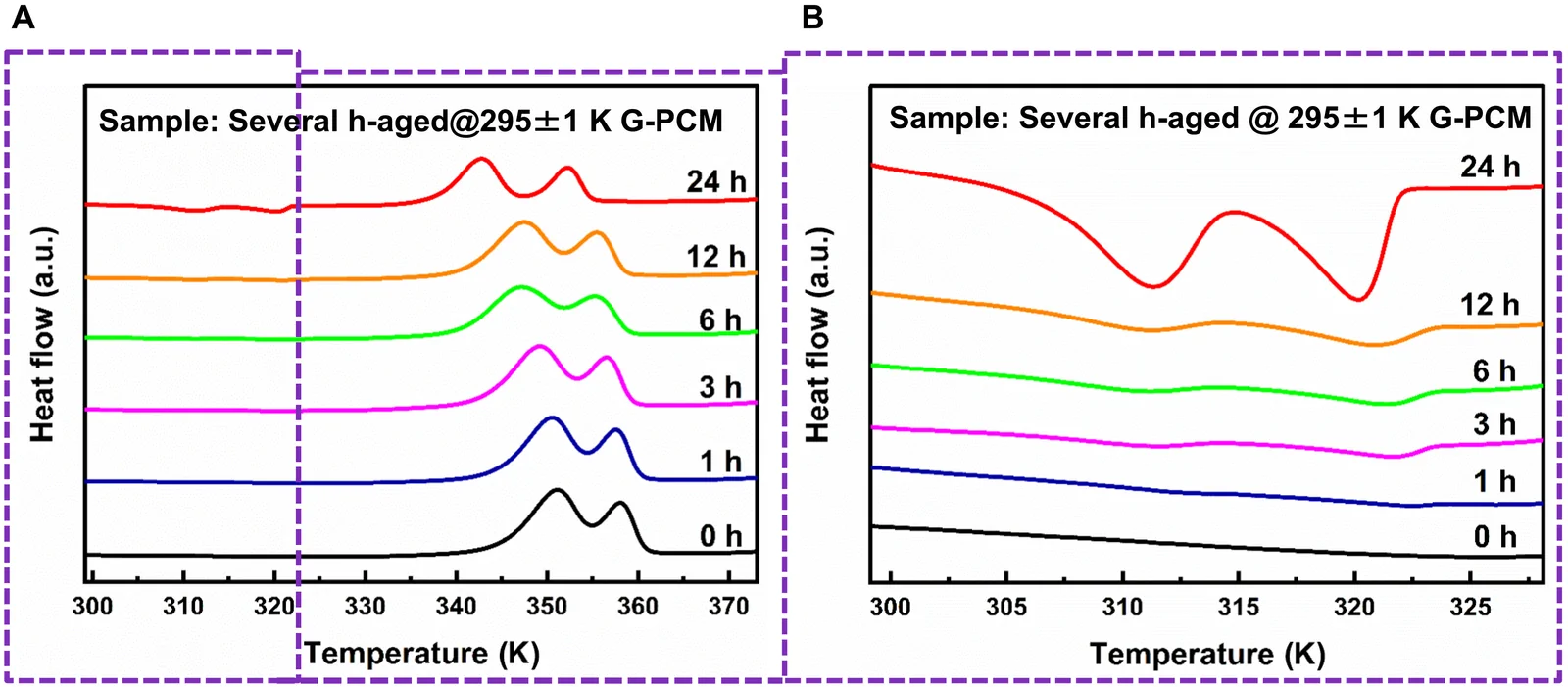
Effect of short isothermal aging at 298 K on the reheating response of the G-PCM. (**A**) DSC curves after 0 to 24 hours holds at 298 K under Ar, following melting and vitrification. (**B**) Enlarged view of the 300- to 325-K region.

Figure 5 (A and B) shows reheating DSC curves after isothermal holds of 0 to 24 hours at 298 K within the DSC under Ar. Each specimen was first melted at 453 K, cooled to 273 K, held for 10 min to form the amorphous state, and then brought to 298 K for aging. With increasing hold time, the glass transition/enthalpy recovery region evolved, and features near 308 to 317 K became more pronounced, while the subsequent cold crystallization event shifted to lower temperature. These trends are consistent with structural/enthalpy relaxation and local rearrangement during the hold. They do not, however, establish that both features are independent glass transitions or prove the formation of microcrystals; preexisting nuclei, subvisible crystallization, and compositional changes of the residual amorphous fraction cannot be distinguished by these DSC scans alone.

**Fig. 6.**
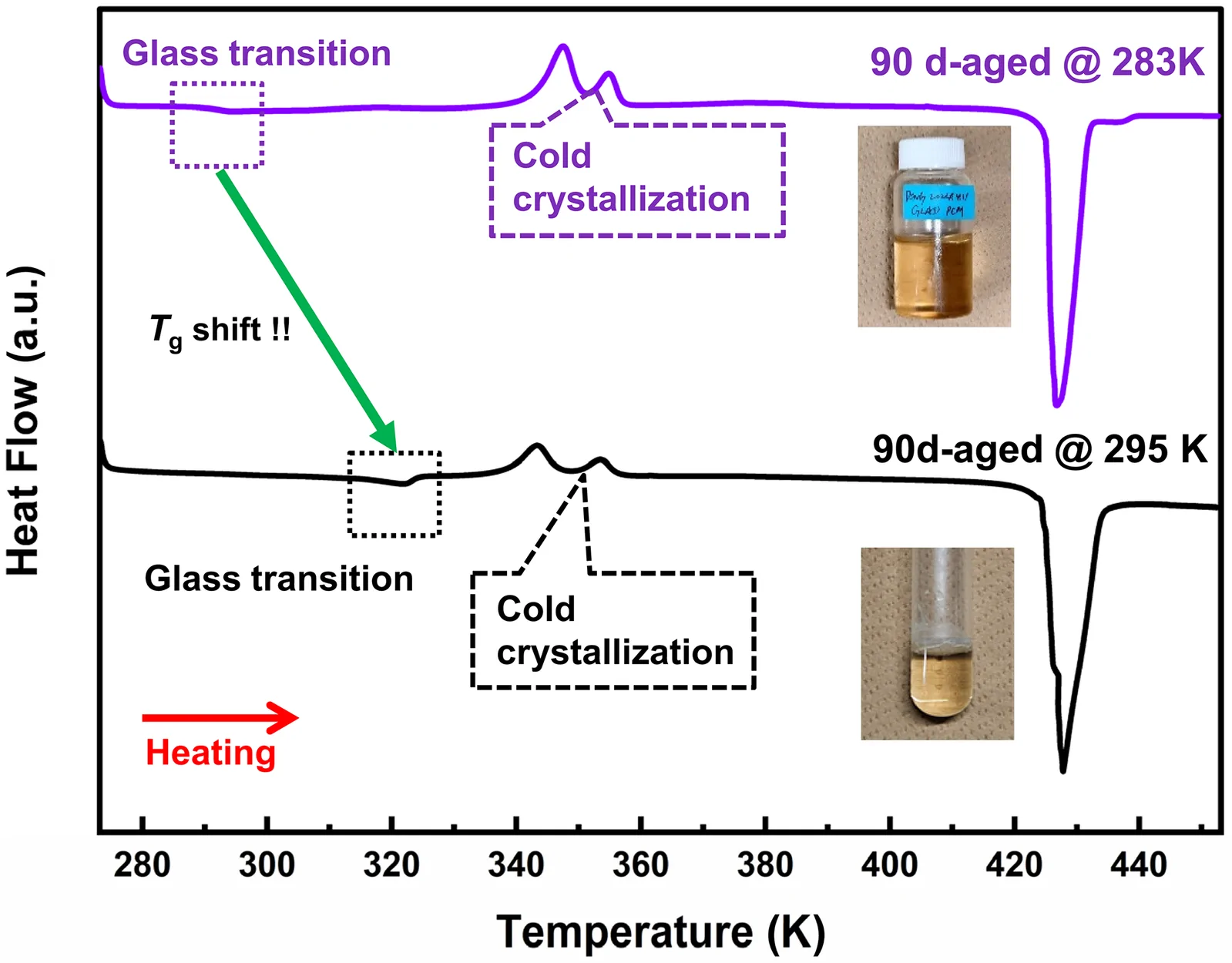
Effect of storage temperature on the 90-day reheating response of the G-PCM. DSC curves of specimens stored at 283 or 295 K under the stated laboratory conditions.

Figure 6 compares reheating DSC curves after 90 days of storage at 283 and 295 K. The specimen stored at 283 K retained an apparent *T*_g_ near 288 K, whereas the specimen stored at 295 K showed an apparent *T*_g_ near 317 K together with an enthalpy recovery feature. The dependence on storage temperature and time is consistent with structural/enthalpy relaxation of the amorphous fraction, but calorimetric *T*_g_ remains an operational reheating quantity rather than a crystallization-free storage boundary (*35*, *49*–*56*). Storage at 298, 303, and 308 K for 30 days further separates relaxation from macroscopic devitrification (figs. S6 and S7). The specimen stored at 298 K remained transparent and retained an amorphous DSC signature. At 303 K, the loss of transparency together with a residual glass-transition feature is consistent with coexistence of devitrified and amorphous fractions. At 308 K, opacity, brittleness, and the absence of a detectable glass transition feature are consistent with extensive devitrification. A residual *T*_g_ signal therefore describes only the remaining amorphous material and does not establish whole-sample stability.

The storage limits are consequently duration specific. Under the investigated preparation, sample geometry, and environmental conditions, the onset of macroscopically evident devitrification over 30 days is bracketed between 298 and 303 K. For ∼180 days, 295 ± 1 K is the highest temperature demonstrated in the tested specimen, which retained *L*_c_ = 113.64 J g^−1^. We therefore suggest *T*_s_ ≤ 295 K as a provisional, condition-specific guideline for approximately seasonal storage. The 298 K result is limited to 30 days, and storage at or above 303 K should be avoided over monthly timescales under comparable conditions. A quantitative boundary requires replicated isothermal experiments and a time-temperature-transformation diagram.

**Fig. 7.**
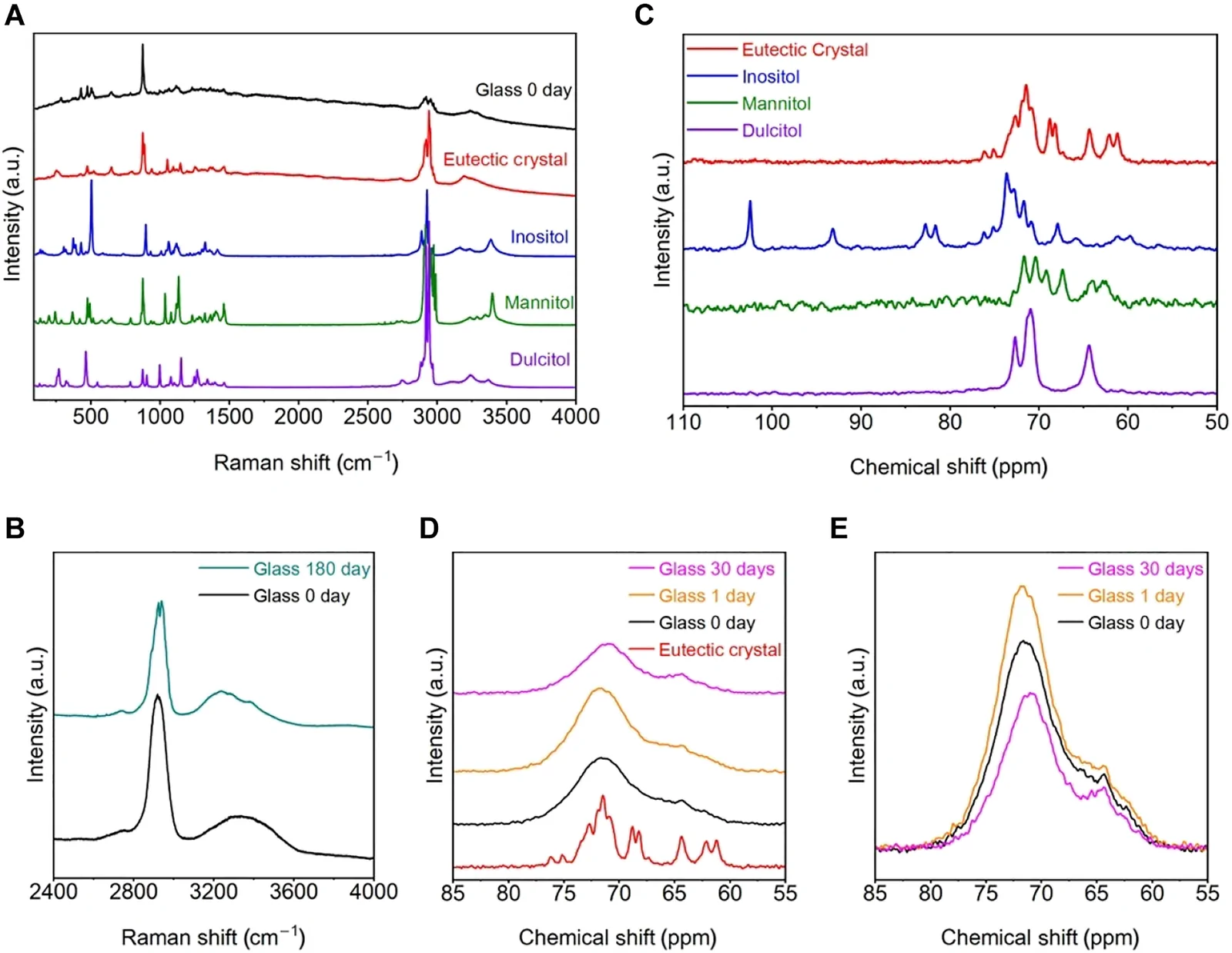
Raman and solid-state NMR characterization of the G-PCM and its constituent sugar alcohols. (**A**) Raman spectra of the as-prepared glassy G-PCM (0 days); eutectic crystalline G-PCM; and crystalline mannitol, dulcitol, and inositol. (**B**) Enlarged Raman spectra in the 2400- to 4000-cm^−1^ region of the glassy G-PCM before and after storage at 295 ± 1 K under laboratory air for 180 days. (**C**) NMR spectra of the eutectic crystalline G-PCM and the crystalline individual components. (**D**) NMR spectra of the eutectic crystalline G-PCM and glassy G-PCM aged at 298 K for 0, 1, and 30 days. (**E**) Overlaid glassy-state spectra from (D), shown over the expanded chemical shift range of 55 to 85 ppm to facilitate the comparison of aging-induced spectral changes.

The quantitative reanalysis of the Raman and ^13^C CPMAS solid-state nuclear magnetic resonance (NMR) spectra is provided in the Supplementary Materials (tables S3 and S4). In the 200- to 1600-cm^−1^ Raman fingerprint region (Fig. 7A), the fresh- and 180-day glass spectra remained highly similar (aligned Pearson *r* = 0.944), while the aged spectrum was more similar to the eutectic-crystal reference (*r* = 0.899) than was the fresh glass (*r* = 0.838); the O─H envelope broadened (Fig. 7B), but its maximum did not exhibit a preprocessing-robust shift. The dominant NMR envelope (Fig. 7, D and E) narrowed from 6.24 to 5.58 parts per million (ppm) over 30 days and remained highly similar after global alignment (*r* > 0.996), without new resolved sharp crystalline resonances. These modest changes are consistent with limited redistribution of local environments and a possible local crystal-like contribution while preserving a predominantly disordered bulk structure; DSC enthalpy recovery remains the primary evidence for structural/enthalpy relaxation.

Prior studies have similarly reported aging-dependent changes in calorimetric *T*_g_ (*54*, *55*). Structural relaxation can lower configurational enthalpy and alter apparent *T*_g_, but relaxation and crystal nucleation and growth remain parallel, mobility-dependent processes (*56*). Accordingly, the apparent *T*_g_ of 317 K after aging characterizes the amorphous material detected during reheating, not a whole-sample storage ceiling; the quantitative spectroscopic limitations are discussed in the Supplementary Materials.

The G-PCM is charged by melting near 423 K and releases stored heat by cold crystallization near 346 K. Recharging therefore requires a heat source capable of reaching at least the melting temperature, such as suitable solar-thermal collectors or industrial waste-heat streams. Industrial heat below 150°C represents a substantial fraction of process demand and waste-heat availability (*57*), so the transition-temperature range is potentially useful. Translation to a system nevertheless requires heat-exchanger design, control of the cold crystallization trigger, and compatible sealed containment.

The as-prepared melting enthalpy *L*_m_ = 248.1 J g^−1^ and density of 1.49 kg liter^−1^ correspond to a total fusion-energy density of ∼370 kJ liter^-1^. The heat measured as retrievable cold crystallization enthalpy is lower: *L*_c_ = 156.5 J g^−1^ (∼233 kJ L^−1^) for the fresh glass and 113.64 J g^−1^ (∼169 kJ L^−1^) after 180 days at 295 ± 1 K. Comparisons with solid-solid heat storage ceramics or phase change alloys (*58*–*60*) must therefore distinguish total fusion enthalpy from the cold crystallization heat actually retained after storage and must use comparable cycling and aging protocols.

In essence, these results establish a proof of concept for storing solid-liquid transition enthalpy in a vitrified, solid-like charged state and releasing part of that enthalpy by cold crystallization. The concept broadens the design space beyond conventional and supercooled-liquid PCMs, but further development requires replicated time-temperature-transformation measurements, controlled humidity and controlled oxygen aging, chemically compatible encapsulation, device-scale heat transfer validation, and repeated long-duration storage rejuvenation testing. Efficient triggering and recovery of the cold crystallization heat also remain engineering priorities.

## MATERIALS AND METHODS

### Materials

d-Mannitol (99.0% purity, *T*_m_ = 440 K, Kao, Tokyo, Japan), dulcitol (99.0% purity, *T*_m_ = 460 K, FUJIFILM Wako Pure Chemical, Osaka, Japan), and myo-inositol (99.0% purity, *T*_m_ = 497 K, FUJIFILM Wako Pure Chemical) were mixed and melted in air in a mannitol/dulcitol/inositol mass ratio of 0.557:0.237:0.206. Subsequently, the mixture was cooled naturally to room temperature and reheated at 373 K to form the crystalline ternary eutectic system.

### Selection of the ternary composition

The ternary formulation was established during an earlier composition-screening campaign using combined experimental thermal analysis and thermodynamic guidance. Multiple binary and ternary mannitol-dulcitol-inositol compositions were prepared gravimetrically and examined under a common thermal analysis protocol. The composition-dependent melting and crystallization behavior was evaluated from the heating and cooling curves.

The final formulation was selected from the compositions examined using two operational criteria: (i) the lowest reproducible apparent melting temperature and (ii) the widest crystallization-free supercooling interval, corresponding to the greatest resistance to crystallization during cooling. The mannitol/dulcitol/inositol mass ratio of 0.557:0.237:0.206 provided the most favorable combination of these properties among the tested formulations and was therefore identified as the ternary eutectic glass-forming composition within the resolution of the original screening.

Because thermodynamic expressions use mole fractions rather than mass fractions, the mole fraction of component i was calculated as

$$X_{i}=\frac{w_{i}/M_{i}}{\sum_{j}w_{j}/M_{j}}$$

where $w_{i}$ and $M_{i}$ are the mass fraction and molar mass of component i, respectively. *j* is the summation index of all components (*j* = *M*, *D*, *I*) The experimentally used mass fractions $w_{M}=0.557$, $w_{D}=0.237$, and $w_{I}=0.206$ correspond to mole fractions of approximately

$$X_{M}=0.5557,X_{D}=0.2365,X_{I}=0.2078$$

Here, the subscripts M, D, and I denote mannitol, dulcitol, and inositol, respectively. A detailed discussion according to thermodynamic interpretation is included in the Supplementary Materials.

### Thermal analysis

The *T*_m_, *T*_c_, *L*_m_, *L*_c_, and *T*_g_ values of the G-PCM were measured by DSC (DSC 3+, Mettler Toledo, Greifensee, Switzerland) using Al crucibles under an Ar atmosphere. Before each scan rate experiment, samples were held at 453 K for 60 min to obtain complete melting and erase previous thermal history. Conventional DSC was conducted over 243 to 453 K at matched cooling and subsequent heating rates of 1, 2, 5, 10, and 50 K min^−1^. In a separate series used to determine the heating rate dependence of *T*_g_ for the Adam-Gibbs-Vogel (AGV) analysis, identically prepared samples were heated from 243 to 453 K at 0.5, 10, 15, 20, 25, 35, 40, 45, 50, or 100 K min^−1^. The heat capacities of the amorphous and crystalline G-PCM were measured by temperature-modulated DSC using a modulation amplitude of ±0.5 K, a period of 50 s, and an underlying heating rate of 1 K min^−1^.

Flash-DSC measurements were performed using a Flash DSC 2+ instrument (Mettler Toledo, Greifensee, Switzerland) under an Ar flow of 80 ml min^−1^. In the cooling-history protocol, the molten sample was cooled at *q*_c_ = 0.1, 0.2, 1, 2, 5, or 10 K s^−1^ and reheated at a fixed *q*_h_ of 0.1 K s^−1^ (Fig. S4). In the heating-rate protocol, *q_c_* was fixed at 10 K s^−1^ and *q*_h_ was varied over 0.1, 0.2, 1, 2, 5, and 10 K s^−1^ (fig. S5). For the Flash-DSC series, the reported transition temperatures correspond to the apparent onset of the glass-transition baseline step (*T*_g,onset_), the onset of the cold crystallization exotherm (*T*_c,onset_), and the melting-peak temperature (*T*_m,peak_). When cold crystallization could not be resolved independently, no separate *T*_c,onset_ or crystallization enthalpy was assigned.

### Characterizations

The crystalline structure of the G-PCM was determined using XRD (SmartLab with a Reactor X heating module, Rigaku, Tokyo, Japan) at different temperatures. The G-PCM crystalline powder was heated from 295 to 453 K for melting and then cooled to 288 K. Subsequently, the sample was reheated to 373 K for cold crystallization, and the heating and cooling rates were maintained at 10 K·min^−1^. XRD was performed at 295 K (crystalline), 453 K (liquid), 373 K (SCL), 288 K (amorphous), 323 K (SCL), and 373 K (cold crystallization) using an α-Al_2_O_3_ sample holder. Raman spectroscopy of the samples was performed using a Raman microscope (XploRA, HORIBA, Kyoto, Japan) equipped with a 532-nm laser, and an attenuator filter was used to reduce the laser power to 0.1% of the incident power to avoid sample damage. The samples were then placed on glass slides and examined under a 100× objective lens. ^13^C CPMAS SS-NMR spectroscopy was conducted using a 500-MHz NMR spectrometer with magic angle spinning at 10 kHz (Avance Neo 500, rotor size: φ 3.2 mm, Bruker, Billerica, MA, USA). Mannitol, inositol, and dulcitol crystal powders and G-PCM samples in their crystalline states were analyzed at 298 K. The as-prepared glassy G-PCM was analyzed at 283 K, in addition to those aged at 298 K for 1 and 30 days.

### Durability and long-term heat storage studies

The G-PCM was subjected to durability studies using DSC to simulate practical heat storage and release cycles. It was heated to 453 K and then cooled to 243 K at 10 K·min^−1^ for 100 cycles. The thermophysical properties of the G-PCM were measured before and after the entire melting and solidification cycle using a heating/cooling rate of 2 K·min^−1^.

For the 180-day storage test, the G-PCM was melted, cooled to form the amorphous state, placed in a petri dish, and stored at 295 ± 1 K under laboratory air. The specimen was not protected by an inert-gas atmosphere or hermetic encapsulation; relative humidity was neither controlled nor recorded, and water uptake and oxygen exposure were not quantified. Short-time aging for 0 to 24 hours was performed in the DSC at 298 K under Ar after melting at 453 K and cooling to 273 K. Separate storage experiments were conducted for 90 days at 283 and 295 K and for 30 days in incubators at 298, 303, and 308 K. After storage, thermal transitions were measured by DSC under the conditions specified in the corresponding figure captions. Optical photographs and SEM (JSM-7001FA, JEOL, Tokyo, Japan) were used to examine macroscopic appearance and morphology. The uncontrolled air test should not be interpreted as a controlled humidity resistance or oxidation experiment.

## Supplementary Materials

### This PDF file includes:

Tables S1 to S4 Figs. S1 to S9 References
