# Supplementary material for: Third-generation ultralong-term latent heat storage glass based on a sugar alcohol mixture
Source: Sci Adv. 2026 Sep 23;12(39):eaei4311. doi: 10.1126/sciadv.aei4311 (PMC13600243; doi:10.1126/sciadv.aei4311)
Supplement: Supplementary file 1 [file sciadv.aei4311_sm.pdf]

Science Advances
AAAS

Supplementary Materials for

Third-generation ultralong-term latent heat storage glass based on a sugar alcohol mixture

Kaixin Dong *et al.*

Corresponding author: Takahiro Nomura, nms-tropy@eng.hokudai.ac.jp

Sci. Adv. **12**, eaei4311 (2026)
DOI: 10.1126/sciadv.aei4311

This PDF file includes:

Tables S1 to S4
Figs. S1 to S9
References

Operational distinction among latent-heat-storage concepts

In this study, "1st-," "2nd-," and "3rd-generation" denote an operational classification based on the charged PCM state and its heat-retention and release pathways. Conventional PCMs store liquid above T_m; supercooled-liquid PCMs retain a metastable liquid below T_m by suppressing premature crystallization; and G-PCMs are cooled through T_g into a kinetically arrested amorphous solid and release heat by cold crystallization on reheating. Vitrification reduces mobility but does not ensure indefinite stability because relaxation and devitrification remain time- and temperature-dependent. Table S1 summarizes these distinctions and differentiates the present additive-free ternary eutectic from earlier matrix-stabilized systems (*14, 29, 33, 35, 61*).

Table S1. Operational comparison of the three latent-heat-storage concepts used in this study.

Feature	Conventional liquid PCM ("1st generation")	Supercooled-liquid PCM ("2nd generation")	Glassy PCM ("3rd generation")
**Defining charged state**	Liquid above T_m (2, 3, 8)	Metastable liquid below T_m, without vitrification (13, 17, 26, 30, 34)	Kinetically arrested amorphous solid formed by cooling through T_g (14, 29, 33, 35, 61)
**Heat-retention basis**	Thermal insulation limits cooling and solidification while the PCM remains above its transition range	Premature nucleation and crystal growth are suppressed in the metastable liquid	Vitrification reduces molecular mobility; lifetime remains governed by relaxation and crystallization kinetics
**Heat-release pathway**	Cooling and solidification near T_m	Deliberately triggered nucleation and crystallization, or temperature-induced crystallization	Reheating through T_g into the cold-crystallization range near T_c
**Principal failure or loss pathway**	Heat leakage causes unintended cooling or solidification; leakage from the liquid state is possible	Premature crystallization initiated by surfaces, impurities, seeds, or mechanical perturbations	Structural/enthalpy relaxation and devitrification outside the condition-specific time–temperature window
**Handling and containment**	The charged state is liquid; liquid-compatible containment is required	The charged phase remains molecularly liquid; containment and trigger management remain necessary	Solid-like, nonflowing charged state; containment remains necessary during melting and for environmental protection
**Representative engineering approach***	Insulation, heat-exchanger design, encapsulation, and support structures (2, 3, 8)	Composition, purity, and interface control; optional stabilizers; engineered triggers (13, 17, 30, 34)	Glass-forming composition and cooling protocol; earlier polymer or ionic matrices (14, 29, 33); present G-PCM additive-free
**Key design and validation metrics**	T_m, fusion enthalpy, storage density, heat-transfer rate, insulation, containment, cycling stability	Supercooling, induction time, nucleation probability, trigger reliability, growth rate, retained capacity	T_g, T_c, R_c, τ(T), induction time, retained L_c after aging, temperature–time–environment stability

**The ordinal labels are operational, not a universal taxonomy or performance ranking. Additives are formulation choices, not generation-defining. T_m, melting temperature; T_g, calorimetric glass-transition temperature; T_c, cold-crystallization temperature; R_c, critical cooling rate; $\tau(T)$, structural-relaxation time. Because calorimetric T_g depends on scan rate and thermal history, it does not alone define a crystallization-free storage ceiling.*

Thermodynamic interpretation of composition-dependent melting

The composition dependence of the liquid-phase stability was interpreted using the Gibbs free energy of a nonideal liquid mixture:

$$G^L = \sum_i X_i\, G_i^{\circ,L} + RT \sum_i X_i \ln X_i + G^E, \tag{S1}$$

where X_i is the mole fraction of component i, $G_i^{\circ,L}$ is the Gibbs free energy of pure liquid component i, R is the gas constant, T is the absolute temperature, and G^E is the excess Gibbs energy. The first two terms describe the pure-component and ideal-mixing contributions, respectively, whereas G^E accounts for nonideal intermolecular interactions.

A Redlich–Kister-type substitutional regular-solution expression containing binary and ternary interaction terms was used as the modeling framework:

$$G^E = \sum_{i<j} \Omega_{ij}\,(T) X_i X_j + \Omega_{MDI}(T) X_M X_D X_I, \tag{S2}$$

where Ω_{ij} and Ω_{MDI} are the binary and ternary interaction parameters, respectively. Their temperature dependence may be represented by

$$\Omega_{ij}(T) = A_{ij} + B_{ij}T \tag{S3}$$

and

$$\Omega_{MDI}(T) = C + DT. \tag{S4}$$

For equilibrium between a liquid mixture and pure crystalline component i,

$$\mu_i^L = \mu_i^S. \tag{S5}$$

Neglecting the heat-capacity difference between the liquid and crystalline states, the liquidus relation can be approximated as

$$\ln(X_i\gamma_i) = -\frac{\Delta H_{\mathrm{fus},i}}{R}\left(\frac{1}{T} - \frac{1}{T_{m,i}}\right), \tag{S6}$$

where γ_i is the activity coefficient, $\Delta H_{\mathrm{fus},i}$ is the molar enthalpy of fusion, and $T_{m,i}$ is the melting temperature of pure component i. The activity coefficient is related to the partial molar excess Gibbs energy by

$$RT\ln \gamma_i = \bar{G}_i^E. \tag{S7}$$

These relations were used during the original materials-screening stage to interpret nonideal melting-point depression and to guide the search toward the low-melting ternary composition region.

Durability of the G-PCM

Figure S1 shows the evolution of T_m, T_g, T_c, and the associated enthalpies (L_m and L_c) over 100 DSC heating–cooling cycles. The lack of any systematic drift in these characteristic temperatures and enthalpies indicates that repeated melting, vitrification, and cold crystallization do not measurably degrade the phase-change response under the applied cycling protocol.

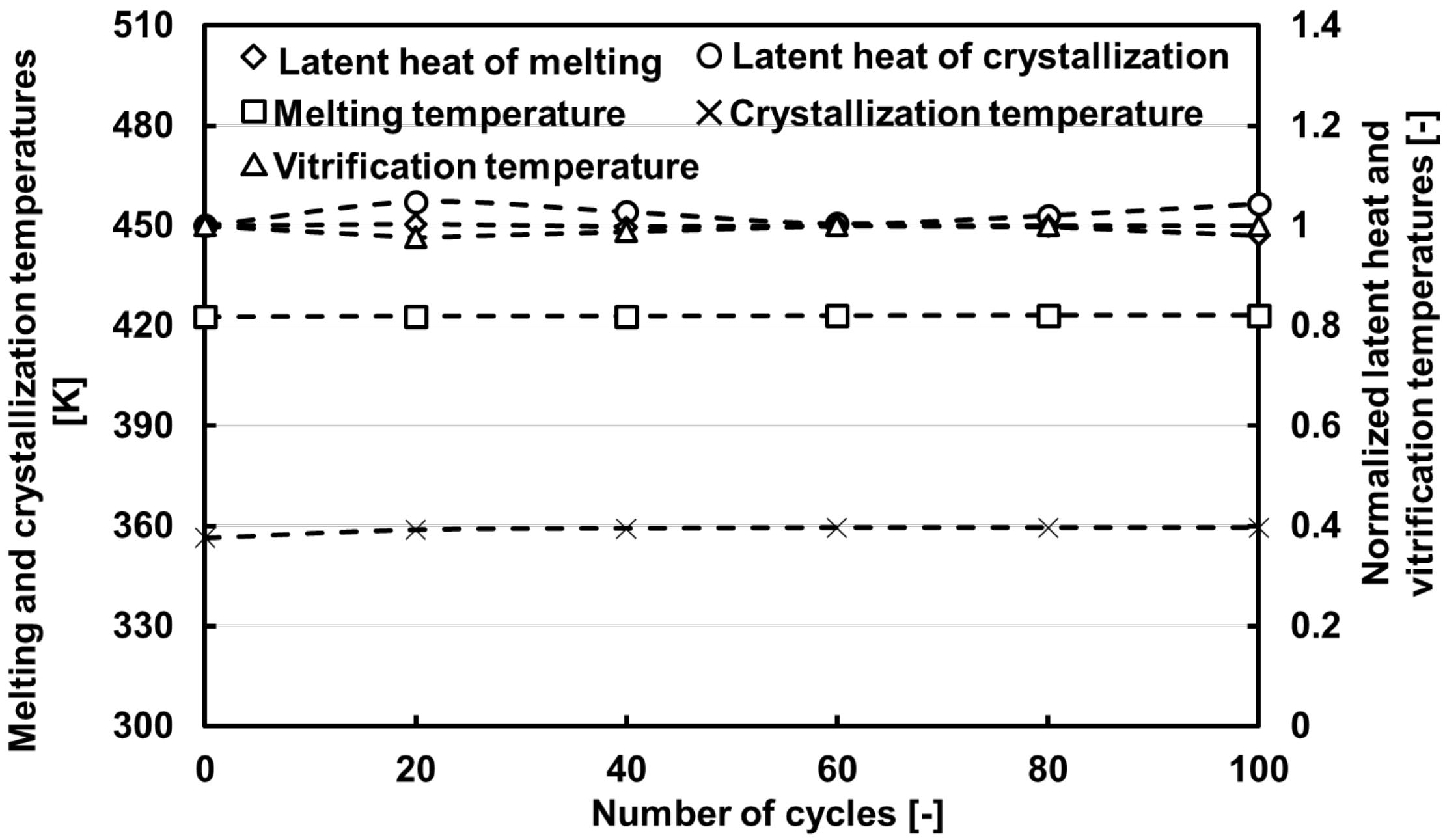

Fig. S1| Thermal cycling stability of the G-PCM. Evolution of the characteristic temperatures (T_m, T_g, and T_c) and latent heats (L_m and L_c) of the G-PCM during 100 DSC heating–cooling cycles (10 K min^{-1}, 243–453 K [−30 to 180 °C], under N_2). T_m and T_c are plotted on the left axis, while T_g, L_m, and L_c are normalized to their initial values (right axis).

Calculation of relaxation time of the G-PCM using the Adam–Gibbs–Vogel (AGV) equation

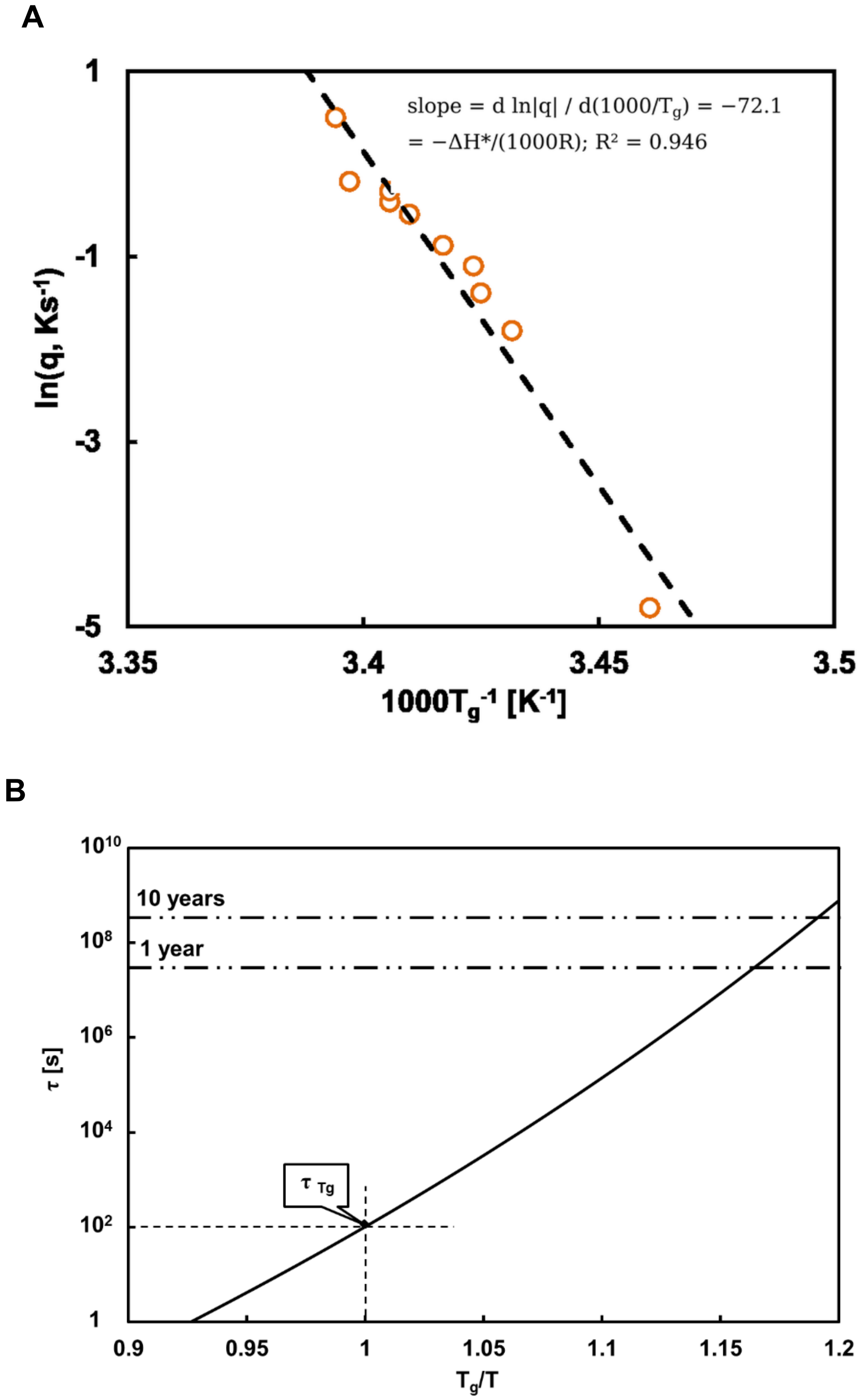

Fig. S2| Determination of fragility and relaxation time of the G-PCM. (**A**) Heating-rate dependence of the apparent glass-transition temperature: ln(q) plotted against 1000/Tg with least-squares fitting. The fitted slope of −72.1 equals $-\Delta H^*/(1000R)$. (**B**) Temperature dependence of the mean structural-relaxation time, τ, estimated using the AGV relation. The reference T_g is defined by $\tau = 100$ s.

Glassy (amorphous) materials are thermodynamically out of equilibrium and may evolve toward a lower-enthalpy nonequilibrium state depending on their thermal history. This time-dependent evolution is commonly referred to as structural relaxation. In the present G-PCM, structural relaxation is relevant because it (i) changes the apparent glass transition features (e.g., T_g shift and enthalpy recovery near T_g) and (ii) can reduce the retrievable heat-release enthalpy upon subsequent cold crystallization, even when the sample remains macroscopically glassy.

To quantify the temperature dependence of molecular mobility in the vitrified state, we estimate the mean structural relaxation time (τ) (*45*). Here, τ is used as a kinetic descriptor for the timescale of structural (enthalpy/fictive-temperature) evolution in the glassy state. A longer τ indicates a more "solid-like" glass with slower structural evolution at a given storage temperature. Here, the relaxation time is estimated using the Adam–Gibbs–Vogel (AGV) relation (Eq. S8):

$$\tau = \tau_0 \, exp\left\{ \frac{DT_0}{T\left(1 - T_0 / T_f\right)} \right\} \tag{S8}$$

where D is the fragility/strength parameter, T_0 is an ideal-temperature parameter often associated with vanishing configurational entropy, and T_f is the fictive temperature describing the nonequilibrium structural state. Equivalently, T_f is the temperature at which an equilibrium liquid would have the same configurational state as the nonequilibrium glass. In this analysis, the kinetic fragility index m derived from the heating-rate dependence of T_g is used to calculate D and T_0 as follows:

$$D = 2.303 \frac{\left(m_{min}\right)^2}{\left(m - m_{min}\right)} \tag{S9}$$

$$m_{min} = log\left(\frac{\tau_{T_g}}{\tau_0}\right) \cong log\left(\frac{100}{10^{-14}}\right) = 16 \tag{S10}$$

$$T_0 = T_g\left(1 - \frac{m_{min}}{m}\right) \tag{S11}$$

Here, $\tau(T_g)$ and τ_0 denote the relaxation times at T_g and at the high-temperature limit, respectively, and were assumed to be 100 and 10^{-14} s (*45–48*). The kinetic fragility index m is defined by the slope of log τ with respect to T_g/T at $T = T_g$:

$$m = \frac{d(\log \tau)}{d(T_g / T)}\bigg|_{T=T_g} \tag{S12}$$

$$m \cong \frac{\Delta H^*}{2.303RT_g} \tag{S13}$$

Equation (S12) can be approximated by Eq. (S13) using the apparent activation enthalpy ΔH^*, obtained from the scan-rate dependence of the calorimetric T_g.

$$\frac{d[ln|q|]}{d(1/T_g)} = -\frac{\Delta H^*}{R} \tag{S14}$$

where R and q are the gas constant and heating rate, respectively (*45, 46*).

T_f can be calculated using the following equations:

$$\frac{1}{T_f} = \frac{\gamma_{C_p}}{T_g} + \frac{1-\gamma_{C_p}}{T} \tag{S15}$$

$$\gamma_{C_p} = \frac{C_p^l - C_p^g}{C_p^l - C_p^x} \tag{S16}$$

where $C^l{}_p$, $C^g{}_p$, and $C^x{}_p$ represent the heat capacities of the G-PCM in the equilibrium liquid (SCL), glassy, and crystalline states, respectively (*46*).

Table S2. Symbols and parameters used in the AGV analysis.

Symbol	Unit	Definition	How obtained/used here
T	K	Absolute temperature	Independent variable
T_g	K	Glass transition temperature	Determined by DSC; also used as reference where $\tau(T_g)=\tau T_g$
τ	s	Mean structural relaxation time	Calculated from Eq. (S8)
τ_{Tg}	s	Relaxation time at T_g	Assumed 100 s
τ_0	s	High-temperature limit relaxation time	Assumed 10^{-14} s
D	—	Fragility/strength parameter in AGV relation	Calculated via Eqs. (S9)–(S11) from m
T_0	K	Ideal temperature parameter in AGV (often associated with vanishing configurational entropy)	Calculated via Eqs. (S9)–(S11)
T_f	K	Fictive temperature describing the nonequilibrium structural state of the glass	Calculated from C_p data using Eqs. (S15)–(S16)
m	—	Fragility index describing sensitivity of τ near T_g	Obtained from ΔH^* using Eqs. (S12)–(S14)
ΔH^*	kJ mol^{-1}	Apparent activation enthalpy derived from the Tg–rate dependence	From the slope of ln(q) versus 1000/T_g in Fig. S2(A), including the factor of 1000
R	J mol^{-1} K^{-1}	Gas constant	Constant
q	K min^{-1} (or K s^{-1})	DSC heating rate	Experimental parameter in Eq. (S14) and Fig. S2(a)
C^l_p	J g^{-1} K^{-1}	Heat capacity C_p in equilibrium liquid (SCL) state	Used in Eqs. (S15)–(S16)
C^g_p	J g^{-1} K^{-1}	Heat capacity C_p in glassy state	Used in Eqs. (S15)–(S16)
C^x_p	J g^{-1} K^{-1}	Heat capacity C_p in crystalline state	Used in Eqs. (S15)–(S16)

Estimation of the mean relaxation time of the G-PCM using the AGV equation

To quantify the temperature dependence of molecular mobility in the vitrified G-PCM, the AGV relation was applied using the heating-rate dependence of the apparent T_g. Figure S2A plots $\ln(q)$ against $1000/T_g$. Because the reciprocal-temperature axis is scaled by 1000, the fitted slope of −72.1 equals $-\Delta H^*/(1000R)$, corresponding to $\Delta H^* = 599.5$ kJ mol^{-1}. This value gives a fragility index m = 108.5; the resulting AGV parameters are $D = 6.38$ and $T_0 = 246.2$ K. The value of D indicates relatively fragile behavior near T_g.

The fictive temperatures below T_g were estimated from the heat capacities of the equilibrium liquid, glassy, and crystalline states. The modeled structural-relaxation time τ is shown in Fig. S2B and increases steeply as T_g/T increases, reaching year-scale values within the plotted range. These values describe mean structural/enthalpy-relaxation times under the assumptions of the AGV model and the heat-capacity-based fictive-temperature calculation. They are neither crystallization-induction times nor guaranteed storage lifetimes; experimental storage limits must be established independently for specified temperatures and durations.

Cooling- and heating-rate dependence of G-PCM vitrification and thermal transitions

To assess the sensitivity of vitrification and the measured transition temperatures to the scan rate, the G-PCM was examined using conventional DSC and Flash-DSC. For conventional DSC, the sample was held at 453 K (180ºC) for 60 min to obtain complete melting and erase its previous thermal history, cooled from 453 K (180ºC) to 243 K (-30ºC), and subsequently reheated over the same range at matched rates of 1, 2, 5, 10, and 50 K min^{-1} under Ar. The original 2 K min^{-1} DSC curves are presented in Fig. 2, whereas the additional 1, 5, 10, and 50 K min^{-1} data are shown in Fig. S3.

No exothermic event attributable to crystallization was detected during cooling at any investigated conventional-DSC rate. The subsequent heating scans consistently retained the glass-transition, cold-crystallization, and melting sequence, although the apparent T_g and the position and shape of the cold-crystallization feature varied with scan rate. This behavior is expected because calorimetric T_g is an operational kinetic temperature determined by the relationship between the experimental timescale and the structural-relaxation timescale of the glass-forming liquid (*39–42*).

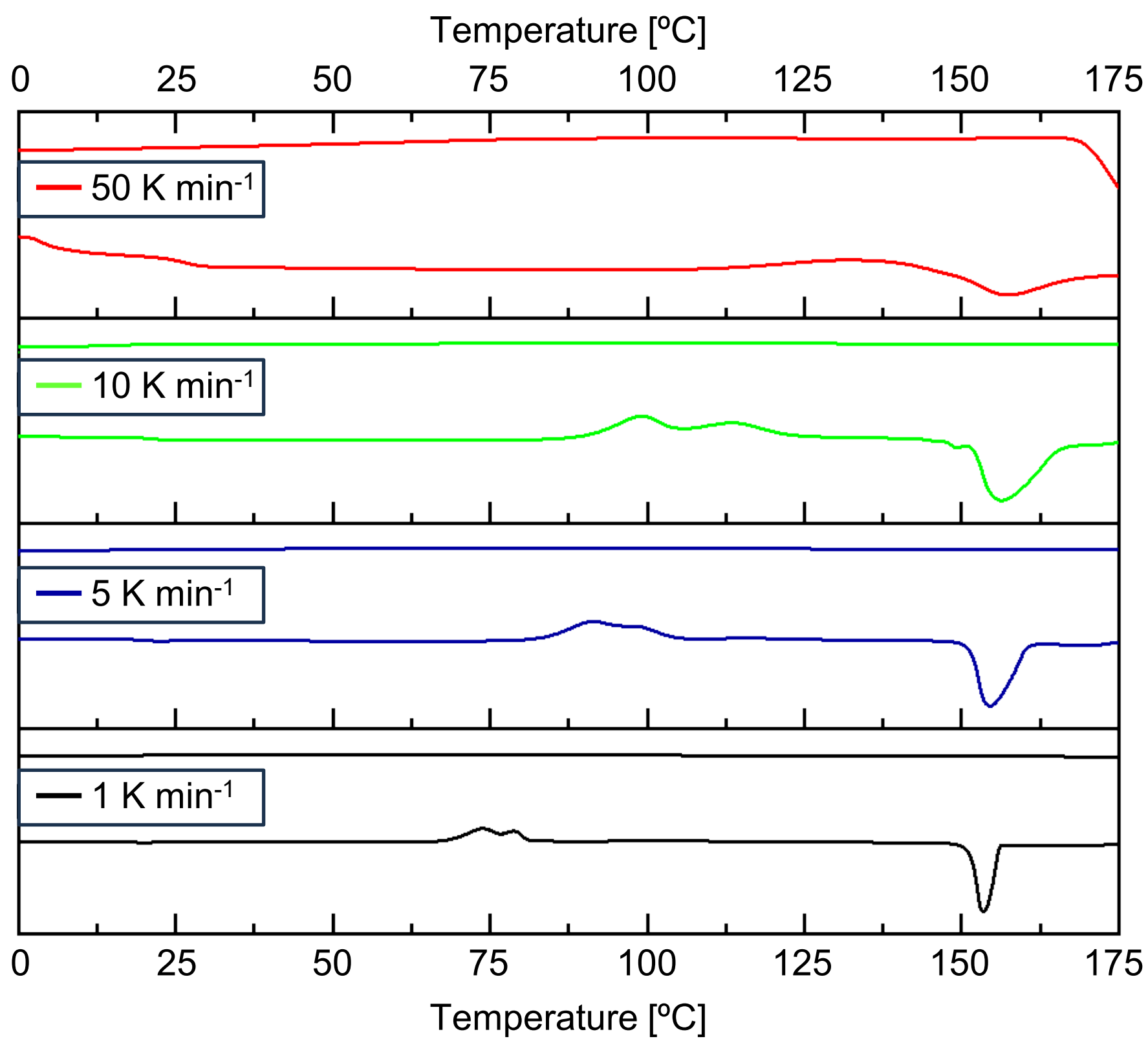

Fig. S3| Conventional DSC thermograms of the G-PCM recorded at matched cooling and subsequent heating rates of 1, 5, 10, and 50 K min^{-1}. Within each rate pair, the upper and lower traces correspond to cooling and subsequent heating, respectively.

Flash-DSC was then used to separate the effects of cooling history and heating rate. In the first protocol, the 453K (180 °C), 5 K s^{-1} molten sample was cooled at q_c = 0.1, 0.2, 1, 2, 5, or 10 K s^{-1} and reheated at a fixed rate of q_h = 0.1 K s^{-1} under an Ar flow of 80 mL min^{-1} (Fig. S4). No crystallization exotherm was detected during cooling. The apparent cooling-branch $T_{g,onset}$ values were distributed within 288–292 K without a resolvable monotonic dependence on q_c; because this small spread can include contributions from dynamic temperature lag and baseline selection, no quantitative cooling-rate dependence is assigned to the cooling-branch $T_{g,onset}$. During the common reheating scan, however, $T_{g,onset}$ increased from 274–277 K after cooling at 0.1–0.2 K s^{-1} to approximately 288–289 K after cooling at rates ≥1 K s^{-1}. This trend is qualitatively consistent with slower cooling producing a more relaxed, lower-fictive-temperature glass, whereas faster cooling freezes a less-relaxed structure (*39–41*). It is not used here as a direct quantitative determination of fictive temperature. Under the common heating condition, $T_{c,onset}$ and $T_{m,peak}$ remained nearly constant at 345–346 K and approximately 422 K, respectively.

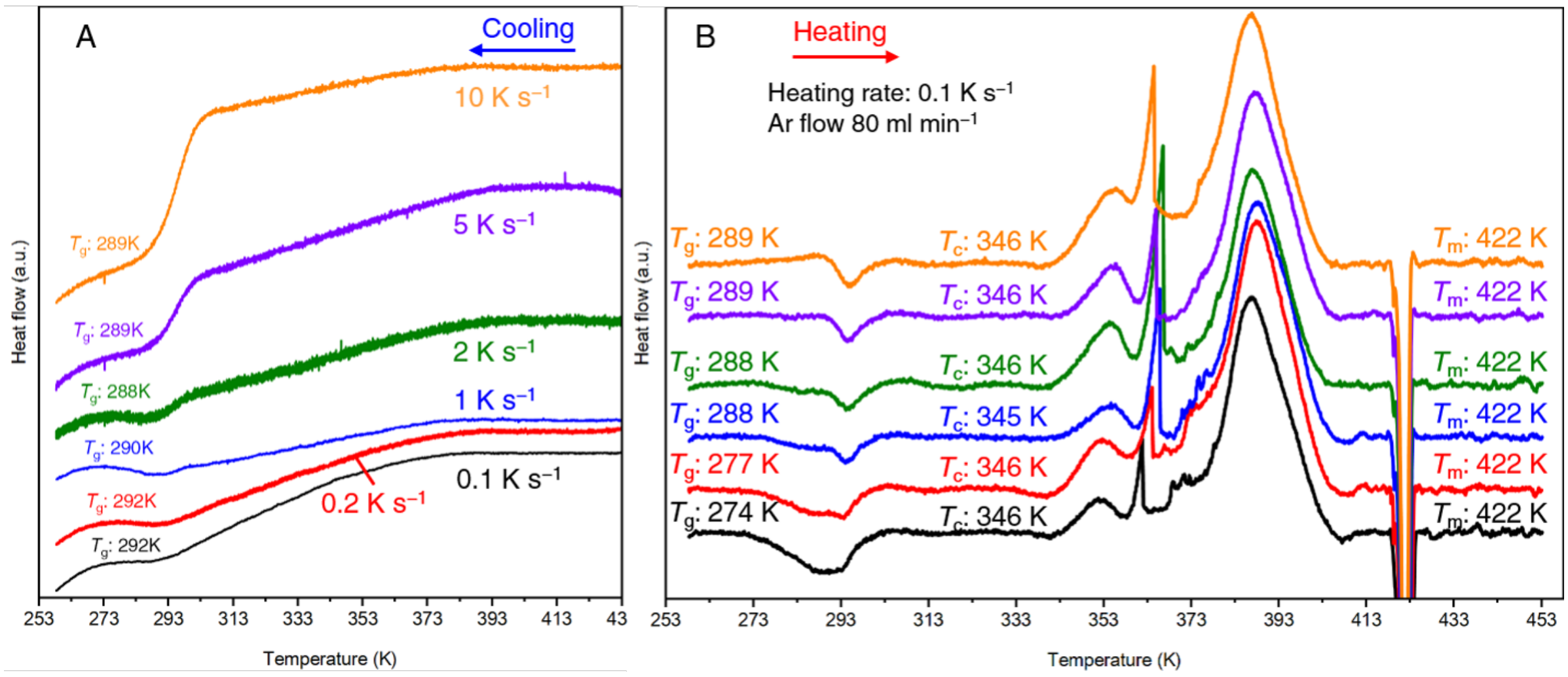

Fig. S4| Cooling-rate dependence of the G-PCM measured by Flash-DSC. (**A**) Cooling thermograms obtained at q_c = 0.1, 0.2, 1, 2, 5, and 10 K s^{-1}. (**B**) Subsequent heating thermograms obtained at a fixed q_h of 0.1 K s^{-1}. Apparent $T_{g,onset}$, $T_{c,onset}$, and $T_{m,peak}$ values are indicated. The arrows denote the scan direction.

In the second Flash-DSC protocol, q_c was fixed at 10 K s^{-1} and q_h was varied from 0.1 to 10 K s^{-1} (Fig. S5). The cooling curves gave an apparent $T_{g,onset}$ of approximately 289 K. During heating, the apparent $T_{g,onset}$ increased overall from 289 to 295 K, while the resolved $T_{c,onset}$ shifted from 346 K at 0.1 K s^{-1} to 367 K at 2 K s^{-1}. Faster heating provides less time for structural relaxation, nucleation, and crystal growth at a given temperature, shifting their apparent occurrence to higher temperatures (*39, 40, 42, 43*). At 5 and 10 K s^{-1}, the cold-crystallization exotherm was not resolved independently from the subsequent thermal response; therefore, no independent $T_{c,onset}$ or crystallization enthalpy was assigned. The apparent $T_{m,peak}$ values lay within 410–422 K but did not vary monotonically with q_h; this spread is therefore not assigned a mechanistic significance and may include dynamic temperature-lag contributions.

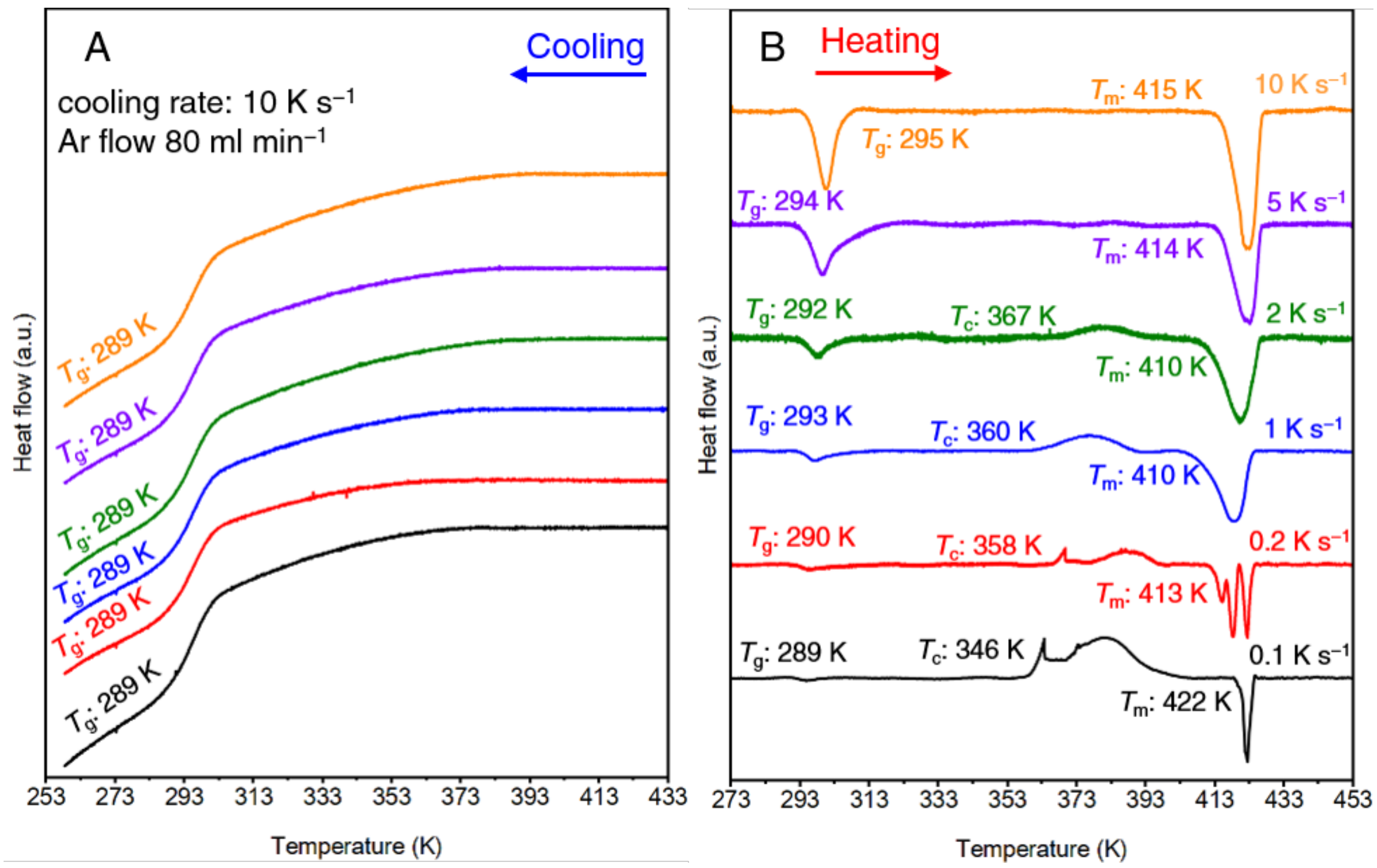

Fig. S5| Heating-rate dependence of the G-PCM measured by Flash-DSC. (**A**) Cooling thermograms obtained at a fixed q_c of 10 K s^{-1}. (**B**) Subsequent heating thermograms obtained at q_h = 0.1, 0.2, 1, 2, 5, and 10 K s^{-1}. The arrows denote the scan direction.

Across the combined conventional- and Flash-DSC experiments, no cooling crystallization was detected over the tested range of 1–600 K min^{-1}. The critical cooling rate, R_c, is the minimum rate required to cool a liquid through its glass-transition region without detectable crystallization (*43, 44*). Because crystallization was absent even at the lowest tested rate, the exact R_c was not reached; instead, the conventional-DSC experiment establishes an operational upper bound of $R_c \leq 1$ K min^{-1} for the milligram-scale specimen under the stated conditions and within the detection sensitivity of the measurement. This result is consistent with strong glass-forming ability and shows that rapid quenching is unnecessary at the DSC sample scale. It should not be interpreted as a bulk-scale critical cooling rate, because increasing sample volume can introduce thermal gradients, longer effective cooling times, and different heterogeneous-nucleation conditions. Bulk implementation therefore requires independent heat-transfer and crystallization validation (*14*).

Long-period heat storage test of the G-PCM

To evaluate the influence of storage temperature on the physical state of the vitrified G-PCM, glassy samples were prepared by heating the G-PCM to 453 K (180 °C) to fully melt and erase thermal history, followed by cooling to form the glassy state. The glassy samples were then stored isothermally in incubators at 298, 303, and 308 K (25, 30, and 35 °C) for 30 days. Photographs of the samples after storage are shown in Fig. S6. The sample stored at 298 K (25 °C) remained transparent and visually similar to the as-prepared glassy state, whereas samples stored at 303, and 308 K (30 and 35 °C) progressively lost transparency with increasing storage temperature; the sample stored at 308 K (35 °C) became opaque/white and brittle, consistent with extensive crystallization during storage.

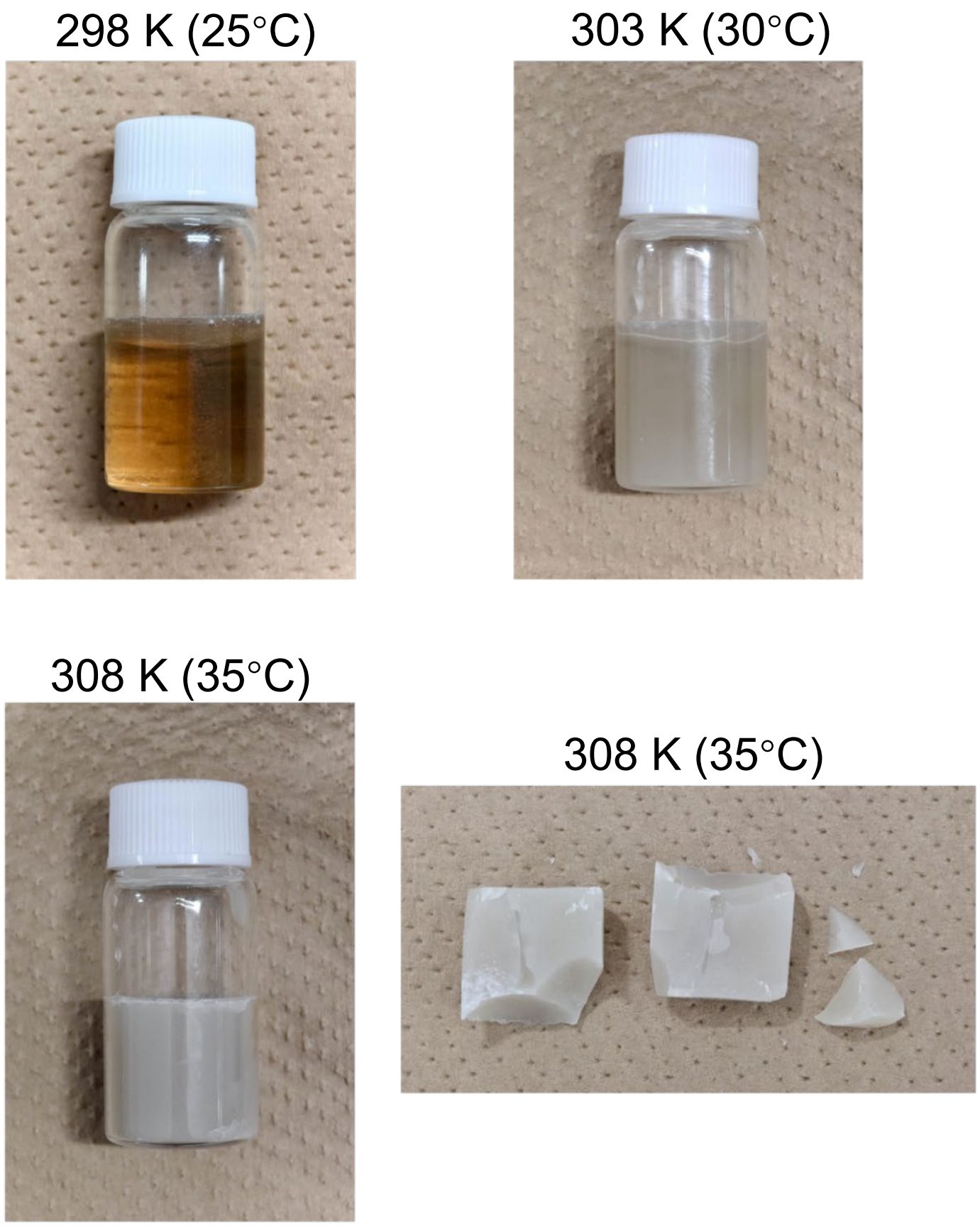

Fig. S6| Photographs of the initially vitrified G-PCM after isothermal storage at 298, 303, and 308 K (25, 30, and 35 °C) for 30 d. Clockwise from the upper left: samples stored at 25 and 30 °C, fractured pieces of the sample stored at 35 °C, and the intact sample stored at 35 °C.

Figure S7 presents the DSC thermograms obtained after storage at 298, 303, and 308 K (25, 30, and 35 °C) for 30 d. During heating from 298 to 323 K (25 to 50 °C) at 1 K min^{-1}, the samples stored at 298 and 303 K (25 and 30 °C) retained detectable glass-transition and enthalpy-recovery features, demonstrating that an amorphous fraction remained after storage. In contrast, no glass-transition feature was detected for the sample stored at 308 K (35°C), consistent with the photographic evidence of extensive crystallization in Fig. S6. During the subsequent cooling from 323 to 273 K (50 to 0 °C), glass-transition features remained detectable for the 298 and 303 K (25 and 30 °C) samples but not for the 308 K (35 °C) sample.

Because 323 K (50°C) is well below the melting temperature of the G-PCM, this limited-temperature cycle does not melt or thermally rejuvenate the crystallized 308 K (35 °C) sample.

A

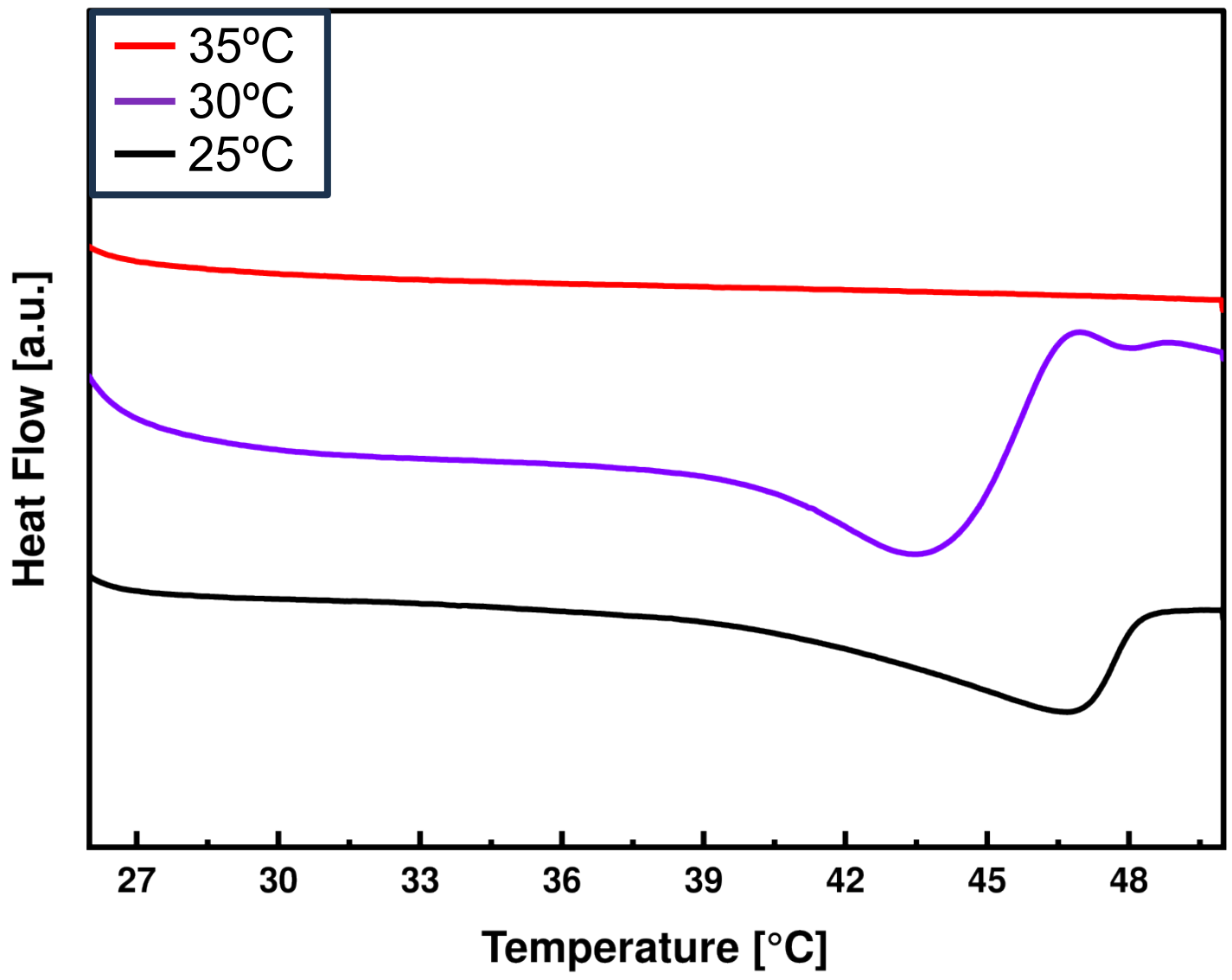

B

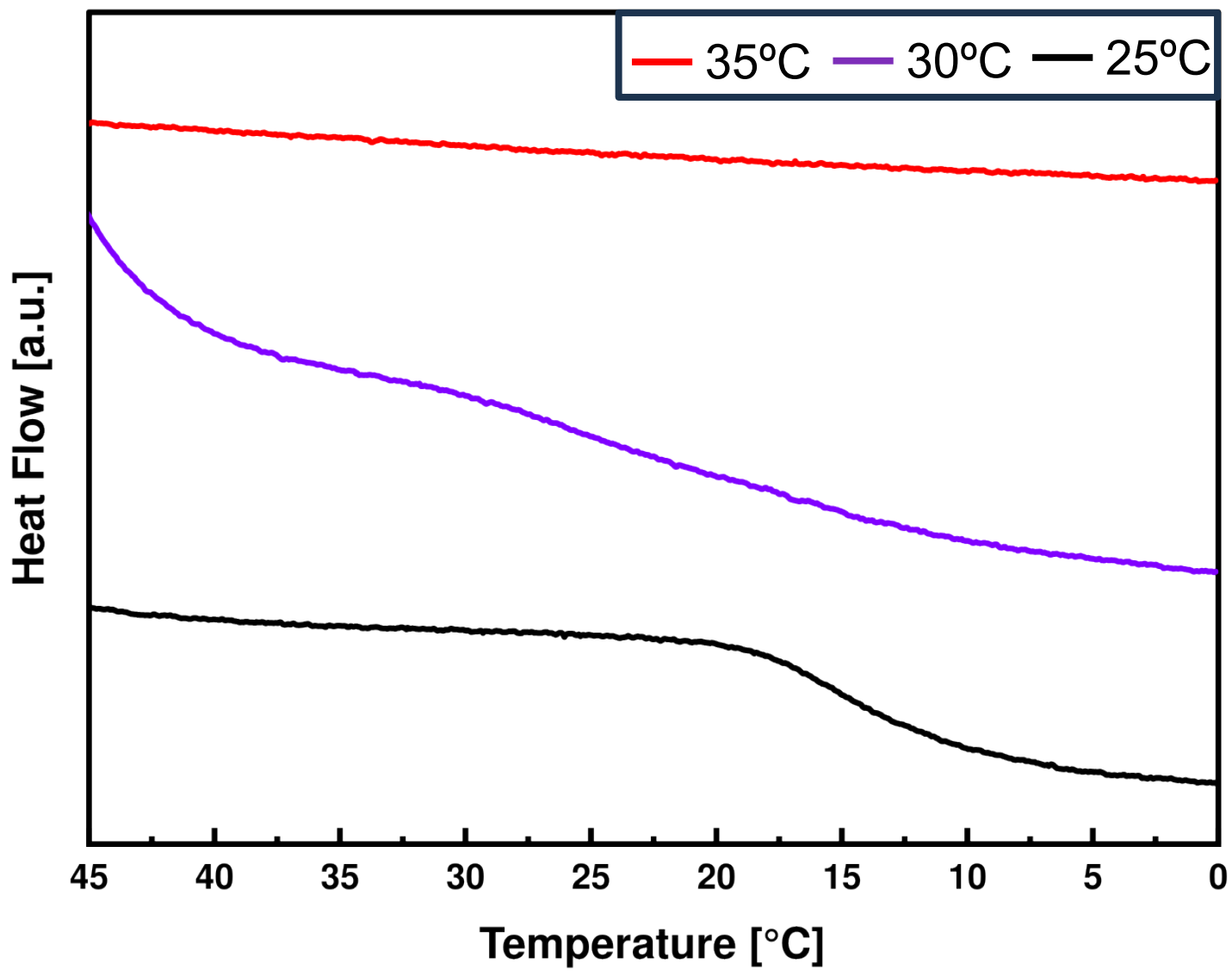

Fig. S7| DSC thermograms of the G-PCM after storage at 298, 303, and 308 K (25, 30, and 35 °C) for 30 d. (**A**) Heating from 298 to 323 K (25 to 50 °C) at 1 K min^{-1}. (**B**) Subsequent cooling from 323 to 273 K (50 to 0 °C) at 1 K min^{-1}.

Interpretation of the apparent glass-transition shift and temperature–time storage window

The 317 K value obtained during reheating after storage at approximately 295 K is an apparent calorimetric glass-transition temperature of the amorphous material present at the time of measurement. Calorimetric T_g is an operational kinetic quantity governed by the relationship between the experimental timescale and the structural-relaxation timescale and depends on scan rate, thermal history, fictive temperature, and structural state; it is therefore neither a thermodynamic phase boundary nor a crystallization-free storage ceiling (*35, 39, 41*). If devitrification is partial, the detected T_g characterizes only the residual amorphous fraction. In this ternary mixture, selective crystallization may additionally change the composition, and hence the T_g, of that fraction. Accordingly, retention of a glass-transition signal after storage at 303 K is compatible with the simultaneous presence of a partially crystallized fraction and does not demonstrate whole-sample stability up to 317 K. An increase in apparent T_g after partial devitrification therefore cannot be attributed exclusively to structural relaxation.

At temperatures above the initial calorimetric T_g of approximately 288 K, structural/enthalpy relaxation of the amorphous phase and crystal nucleation and growth can proceed in parallel because both require molecular mobility. Relaxation lowers configurational enthalpy and rearranges the local molecular environment, whereas the same mobility also permits crystallization. The decrease in the subsequent T_c is consistent with facilitated crystallization but does not establish that relaxation directly generated nuclei; pre-existing nuclei, partial crystallization, and compositional changes in the residual amorphous phase may also contribute. Quantitative spectroscopic evidence and its limitations are evaluated in the following section. Structural relaxation and crystallization are therefore interpreted as coupled, mobility-dependent processes rather than a demonstrated direct causal sequence (*26, 37, 46, 56*).

The combined 90-d tests in (Fig. 6), the 180-d test in (Fig. 4), and Figs. S6 and S7 define duration-specific rather than absolute storage limits. After 30 d, the specimen stored at 298 K remained macroscopically transparent and retained an amorphous DSC signature. At 303 K, loss of transparency together with a residual glass-transition feature is consistent with coexistence of devitrified and amorphous fractions, whereas opacity, brittleness, and the absence of a detectable glass-transition feature after storage at 308 K are consistent with extensive devitrification. Thus, under the investigated preparation, sample geometry, and environmental conditions, the onset of macroscopically evident devitrification over 30 d is bracketed between 298 and 303 K. For approximately 180 d, 295 ± 1 K is the highest temperature demonstrated in the tested specimen, which remained macroscopically transparent and retained L_c = 113.64 J g^{-1}. We therefore propose $T_s \leq 295$ K as a provisional, condition-specific guideline for approximately seasonal storage. The 298 K result is limited to 30 d, whereas storage at or above 303 K should be avoided over monthly timescales under comparable conditions. Storage below the initial T_g of approximately 288 K provides an additional kinetic margin. A quantitative temperature–time boundary requires replicated isothermal-crystallization measurements and a time–temperature–transformation diagram.

Quantitative Raman and 13C CPMAS solid-state NMR analysis

The subtle differences in Fig. 7 were evaluated using model-light line-shape metrics. Common baseline correction, normalization, interpolation onto a common axis, small global-shift alignment, peak-location, positive-area centroid, and half-height FWHM procedures were applied, together with preprocessing-sensitivity tests. Only one spectrum was available for each condition; the sensitivity ranges therefore describe dependence on data treatment rather than specimen-to-specimen uncertainty. Pearson r is used only as a descriptive whole-spectrum similarity index. Because neighboring spectral points are autocorrelated, r is not treated as a significance test or as proof of a phase fraction or mechanism. The 200–1600 cm^{-1} Raman interval was analyzed as the fingerprint region containing the principal skeletal and bending modes, while the strongly overlapping C–H/O–H stretching region was evaluated separately.

In the 200–1600 cm^{-1} Raman fingerprint region, the aligned fresh- and 180-d-glass spectra remained highly similar ($r = 0.944$), indicating retention of the dominant molecular fingerprint. Similarity to the eutectic-crystal reference increased from $r = 0.838$ for the fresh glass to $r = 0.899$ for the 180-d glass. This change is consistent with development of a limited crystal-like vibrational contribution or local ordering in the Raman-probed region, but it neither establishes a bulk crystalline fraction nor identifies nuclei. Absolute phase fractions cannot be recovered from independently normalized, single-location spectra without calibrated response factors and representative spatial mapping.

The O–H stretching envelope was evaluated over 3050–3750 cm^{-1}. Its maximum changed from 3240.6 to 3237.4 cm^{-1}, a nominal displacement of −3.2 cm^{-1}. Under reasonable changes in baseline anchors, signal range, and smoothing window, however, the displacement ranged from −3.2 to +5.9 cm^{-1} and had a median of 0.0 cm^{-1}; a resolved red shift is therefore not assigned. The centroid increased from 3254.6 to 3287.4 cm^{-1} and the FWHM from 116.5 to 255.3 cm^{-1}. Broadening and redistribution of the O–H envelope are thus the reproducible observations. They are compatible with a more heterogeneous distribution of local hydrogen-bonding environments but do not independently prove uniformly stronger hydrogen bonding, reduced free volume, or lower molecular mobility. Unconstrained multipeak decomposition was not performed because the number, positions, and assignments of the strongly overlapping components are not uniquely identifiable from one pre-normalized spectrum.

The ^{13}C CPMAS spectra were analyzed over 55–85 ppm using common baseline correction, smoothing, positive-area normalization, centroid analysis, and half-height FWHM measurement of the dominant 65–80 ppm envelope. Robustness was assessed across 300 combinations of baseline model and region, smoothing window, and spectral-moment range. The centroid values were 70.04, 70.19, and 69.81 ppm for the fresh, 1-d, and 30-d glasses, respectively; the change is small and non-monotonic and is not described as a continuous upfield shift. The corresponding FWHM values were 6.24, 5.92, and 5.58 ppm, representing approximately 5 and 11% narrowing after 1 and 30 d. After small global-shift alignment, the aged spectra remained highly similar to the fresh spectrum ($r > 0.996$). They also remained much broader than the eutectic-crystal reference (FWHM approximately 1.76 ppm), and no new sharp crystalline resonances were resolved.

The NMR narrowing is consistent with a modestly narrower distribution of time-averaged local carbon environments while the bulk remains predominantly disordered. Conventional

CP/MAS intensities depend on polarization-transfer and relaxation efficiencies and were not used to quantify molecular populations or phase fractions. Static CP/MAS spectra also cannot uniquely distinguish hydrogen-bond rearrangement, conformational redistribution, and molecular-mobility changes. In addition, sub-ppm shifts and linewidths can contain acquisition-temperature and cross-polarization contributions; the comparisons therefore assume comparable acquisition conditions and are interpreted descriptively rather than as aging-specific kinetic constants. Direct mobility measurements would require ^{13}C T1 or T1ρ relaxation or variable-contact-time CP experiments.

Overall, the spectroscopy indicates limited local structural evolution without a major transformation of the abundant molecular framework. DSC enthalpy recovery and the thermal-history-dependent apparent T_g remain the primary evidence for structural/enthalpy relaxation. The increase in Raman crystal-reference similarity, the loss of L_c, and the visual changes are complementary evidence consistent with limited local ordering or a small devitrified contribution, but the data do not establish a quantitative causal relationship or crystalline fraction. The NMR series extends only to 30 d and should not be treated as a direct chemical analysis of the 180-d specimen.

Table S3. Quantitative Raman line-shape metrics.

Raman metric	Fresh glass	180-d glass	Eutectic crystal
Fingerprint aligned r versus fresh glass	—	0.944	0.838
Fingerprint aligned r versus 180-d glass	0.944	—	0.899
O–H maximum (cm−1)	3240.6	3237.4	3194.4
O–H centroid (cm−1)	3254.6	3287.4	3255.2
O–H FWHM (cm−1)	116.5	255.3	161.1

The nominal 180-d-minus-fresh maximum shift was −3.2 cm^{-1}, but it varied from −3.2 to +5.9 cm^{-1} across preprocessing choices and had a median of 0.0 cm^{-1}. Pearson r is a descriptive aligned line-shape similarity metric, not a measure of statistical significance or crystalline fraction.

Table S4. Quantitative 13C CPMAS solid-state NMR line-shape metrics.

Sample	Centroid (ppm)	FWHM (ppm)	FWHM change	Aligned r vs fresh
Fresh glass	70.04	6.24	—	—
Glass, 1 d	70.19	5.92	−5%	0.9986
Glass, 30 d	69.81	5.58	−11%	0.9969
Eutectic crystal	69.25	1.76	—	—

One spectrum was analyzed per condition. The values characterize line-shape and preprocessing differences and do not provide replicate-based statistical uncertainty. CP/MAS signal intensity was not treated as directly population-quantitative.

Thermal rejuvenation of the 180-d-aged G-PCM

After storage at approximately 295 K for 180 d, L_c decreased from 156.5 to 113.64 J g^{-1}, a reduction of 27.4%. Together with the increase in apparent calorimetric T_g and the enthalpy-recovery feature near T_g, this decrease is consistent with structural/enthalpy relaxation; contributions from subvisible crystallization, environmental exposure, or chemical change cannot be partitioned quantitatively. The quantitative Raman and NMR analysis above provides complementary evidence of limited local structural evolution while showing that the abundant molecular framework remains predominantly disordered. The 180-d specimen did not exhibit the opaque-white appearance associated with extensive devitrification, but nanoscale ordering or a small crystalline fraction below the measurement sensitivity cannot be excluded (*41, 49–56*).

To evaluate the reversibility of the aging-associated capacity decrease, the 180-d specimen was subjected to two consecutive DSC cycles (Fig. S8). During the first heating scan, it exhibited an apparent T_g of 317 K (44ºC) and L_c = 113.64 J g^{-1}. The specimen was then heated to 453 K (180ºC) to obtain complete melting, cooled using the standard vitrification protocol, and reheated. Complete melting erases the structural-relaxation history and melts any pre-existing crystalline fraction before cooling generates a new glassy state. In the second heating scan, the apparent T_g returned to 289 K (16ºC) and L_c recovered to 148.73 J g^{-1}. The recovered L_c corresponds to 95.0% of the as-prepared value and recovery of 81.9% of the storage-induced enthalpy deficit. The capacity decrease is therefore substantially, but not completely, reversible under the tested melt-reset and revitrification protocol.

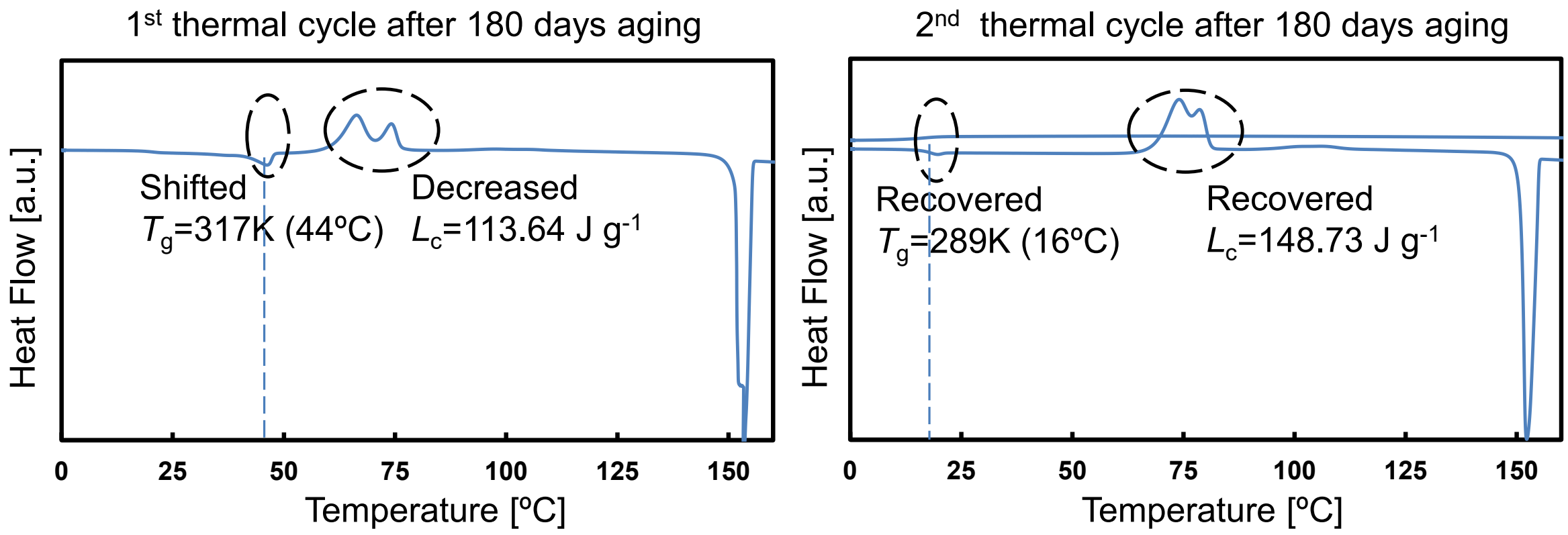

Fig. S8| Thermal rejuvenation of the G-PCM after storage at approximately 295 K (22ºC) for 180 d. Left: DSC curves from the first thermal cycle of the aged specimen, showing an apparent T_g of 317 K (44ºC) and L_c of 113.64 J g^{-1}. Right: DSC curves from the second cycle after heating to 453 K (180ºC) for complete melting, followed by revitrification, showing recovery of T_g to 289 K (16 ºC) and L_c to 148.73 J g^{-1}. Dashed vertical lines mark the apparent glass-transition temperatures, and dashed ellipses highlight the glass-transition/enthalpy-recovery and cold-crystallization regions.

The substantial recovery supports a largely reversible, history-dependent change and argues that irreversible bulk chemical degradation was not the dominant origin of the reduced L_c. Because complete melting also removes any subvisible crystalline fraction, the recovery experiment alone cannot distinguish quantitatively between structural/enthalpy relaxation and partial crystallization. The 100-cycle DSC test (Fig. S1) shows no systematic deterioration in T_g, T_c, T_m, L_c, or L_m during repeated melting, vitrification, and cold crystallization under the investigated laboratory protocol. It does not, however, establish recovery after repeated 180-d storage periods or packaged-device durability.

Environmental exposure, appearance changes, and G-PCM containment

The specimen used for the 180-d storage test was kept at 295 ± 1 K in a Petri dish under laboratory air, without inert-gas protection or hermetic encapsulation. Relative humidity was neither controlled nor recorded, and neither water uptake nor oxygen exposure was quantified. The experiment therefore represents unencapsulated exposure to the encountered laboratory atmosphere, rather than a controlled humidity-resistance test or a defined worst-case condition. Water can plasticize amorphous carbohydrate- and polyol-based materials, lowering T_g and increasing molecular mobility. In the present experiment, however, the apparent T_g increased from 288 to 317 K after 180 d rather than decreasing; this trend does not support substantial bulk water plasticization as the dominant process. Because structural/enthalpy relaxation shifts T_g in the opposite direction, it could mask a smaller moisture-induced shift. Limited water uptake therefore cannot be excluded without controlled-relative-humidity storage and direct water-content measurements.

Two visually distinct changes in appearance should be separated. The cloudy or white appearance of specimens stored at 303 and 308 K for 30 d is primarily consistent with devitrification rather than chemical discoloration (Figs. S6 and S7). Crystallites and crystalline–amorphous interfaces scatter visible light, and cracking of the brittle, extensively devitrified 308 K specimen may add a scattering contribution. This interpretation agrees with the residual amorphous DSC signature at 303 K and absence of a detectable glass-transition feature at 308 K. By contrast, the specimen stored at 295 ± 1 K for 180 d did not become opaque white but exhibited a slight yellow/amber tint (Fig. 4 and Fig. S9); this color change is chemically nonspecific and should not be equated with crystallization.

The initial ternary mixture was prepared by melting in air, and the thermal protocols used 453 K for complete melting and erasure of previous thermal history, above the melting-peak temperature of approximately 423 K. A small concentration of chromophoric species formed during the initial air melt or other high-temperature processing steps therefore cannot be excluded. Chemically plausible pathways include partial oxidation of hydroxyl groups to carbonyl-containing products, dehydration or condensation reactions, and reactions involving trace impurities. Mannitol oxidation and thermal decomposition and dehydration of a related polyol under other conditions have been reported (*62–65)*. These reports establish plausibility only; they do not identify the products or mechanism responsible here. Conventional Maillard browning is not applicable because the formulation contains neither an amino component nor a reducing sugar, and the color change is not assigned specifically to caramelization without chemical-product analysis.

The 180-d Raman comparison and 0–30-d ^{13}C CPMAS NMR series show no major loss of the abundant sugar-alcohol framework within their sensitivity and spectral ranges. They nevertheless cannot exclude low concentrations of carbonyl-containing or conjugated chromophores: the NMR series does not extend to 180 d, the spectra are dominated by abundant aliphatic carbons, and no targeted trace-product analysis was performed. Trace colored products may therefore be visually detectable at concentrations too low to produce resolvable changes in bulk Raman or solid-state NMR spectra.

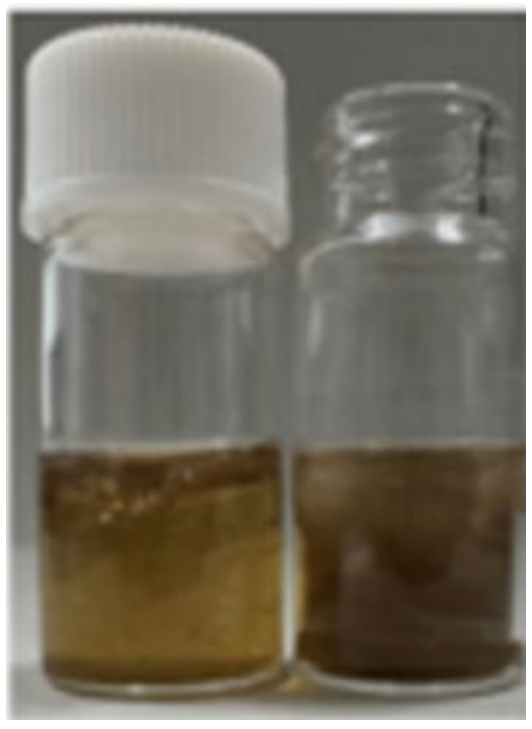

Fig. S9| Representative appearance of unencapsulated G-PCM specimens after storage at 295 ± 1 K for 180 d under laboratory air. Slight discoloration was observed. Relative humidity, sample water content, and oxygen exposure were not controlled or quantified; therefore, the photograph documents macroscopic appearance only and does not establish the chemical origin of the color change.

Appearance alone is not a quantitative measure of thermophysical degradation. During 100 heating–cooling cycles under N_2, T_m, T_c, T_g, L_m, and L_c showed no systematic drift beyond experimental scatter (Fig. S1), indicating a stable bulk phase-change response under that inert-atmosphere protocol. This does not establish stability during 180-d air storage: after unencapsulated storage at 295 ± 1 K, L_c decreased from 156.5 to 113.64 J g^{-1} (27.4%). Structural/enthalpy relaxation, limited devitrification, environmental exposure, and possible chemical change may contribute; the data neither partition these contributions nor quantitatively link yellowing to enthalpy loss. Thermal conductivity and optical absorptance after aging were not measured.

Practical use requires a compatible, leak-tight enclosure because the G-PCM liquefies near $T_m \approx 423$ K. A low-permeability hermetic container with a dry N_2 or Ar headspace should contain the melt, accommodate volume change, and reduce moisture and oxygen exposure, consistent with PCM engineering practice (*2, 14, 29*). It should mitigate—but cannot ensure elimination of—environmental discoloration or degradation because residual oxygen, permeation, impurities, and oxygen-independent reactions may persist. Encapsulation cannot prevent temperature-driven devitrification; controlled-atmosphere aging and cycling must therefore validate the packaged system within the temperature–time storage window.

REFERENCES

1. International Energy Agency, *Renewables 2023: Analysis and forecasts to 2028* (International Energy Agency, 2024); www.iea.org/reports/renewables-2023.

2. G. Wang, Z. Tang, Y. Gao, P. Liu, Y. Li, A. Li, X. Chen, Phase change thermal storage materials for interdisciplinary applications. *Chem. Rev.* **123**, 6953–7024 (2023).

3. J. N. Chiu, J. C. Castro Flores, V. Martin, B. Lacarrière, Industrial surplus heat transportation for use in district heating. *Energy* **110**, 139–147 (2016).

4. A. Kaizawa, H. Kamano, A. Kawai, T. Jozuka, T. Senda, N. Maruoka, T. Akiyama, Thermal and flow behaviors in heat transportation container using phase change material. *Energ. Conver. Manage.* **49**, 698–706 (2008).

5. A. Kaizawa, H. Kamano, A. Kawai, T. Jozuka, T. Senda, N. Maruoka, N. Okinaka, T. Akiyama, Technical feasibility study of waste heat transportation system using phase change material from industry to city. *ISIJ Int.* **48**, 540–548 (2008).

6. A. Kaizawa, N. Maruoka, A. Kawai, H. Kamano, T. Jozuka, T. Senda, T. Akiyama, Thermophysical and heat transfer properties of phase change material candidate for waste heat transportation system. *Heat Mass Transf.* **44**, 763–769 (2008).

7. Q. Ma, L. Luo, R. Z. Wang, G. Sauce, A review on transportation of heat energy over long distance: Exploratory development. *Renew. Sustain. Energy Rev.* **13**, 1532–1540 (2009).

8. T. Nomura, N. Okinaka, T. Akiyama, Waste heat transportation system, using phase change material (PCM) from steelworks to chemical plant. *Resour. Conserv. Recycl.* **54**, 1000–1006 (2010).

9. D. S. Jayathunga, H. P. Karunathilake, M. Narayana, S. Witharana, Phase change material (PCM) candidates for latent heat thermal energy storage (LHTES) in concentrated solar power (CSP) based thermal applications—A review. *Renew. Sustain. Energy Rev.* **189**, 113904 (2024).

10. C. Prieto, L. F. Cabeza, Thermal energy storage (TES) with phase change materials (PCM) in solar power plants (CSP). Concept and plant performance. *Appl. Energy* **254**, 113646 (2019).

11. J. Huang, Y. Zhao, J. Song, J. Chen, K. Wang, Z. Rao, C. N. Markides, Dynamic performance and control of Carnot battery-based combined cooling, heating and power systems with cascaded latent thermal energy stores. *Appl. Energy* **406**, 127286 (2026).

12. Y. Zhao, J. Huang, J. Song, Y. Ding, Thermodynamic investigation of a Carnot battery based multi-energy system with cascaded latent thermal (heat and cold) energy stores. *Energy* **296**, 131148 (2024).

13. X. Shao, Y. Chenxu, B. Wang, N. Zhang, Y. Yuan, Mechanical agitation triggered crystallization of eutectic phase change material xylitol/erythritol with persistent supercooling for controllable heat retrieval. *Sol. Energy Mater. Sol. Cells.* **256**, 112335 (2023).

14. K. Turunen, M. R. Yazdani, S. Puupponen, A. Santasalo-Aarnio, A. Seppälä, Cold-crystallizing erythritol-polyelectrolyte: Scaling up reliable long-term heat storage material. *Appl. Energy* **266**, 114890 (2020).

15. M. Kick, S. Gamisch, S. Gschwander, MD-guided nucleation agent development for supercooling control in fatty alcohol PCM emulsions. *Thermochim. Acta* **752**, 180068 (2025).

16. Y. Zhao, X. Zhang, X. Xu, S. Zhang, Research progress in nucleation and supercooling induced by phase change materials. *J. Energy Storage* **27**, 101156 (2020).

17. S. Yang, X.-F. Shao, H.-Y. Shi, J.-H. Luo, L.-W. Fan, Bubble-injection-enabled significant reduction of supercooling and controllable triggering of crystallization of erythritol for medium-temperature thermal energy storage. *Sol. Energy Mater. Sol. Cells* **236**, 111538 (2022).

18. Y. Liu, X. Li, Y. Xu, B. Fu, C. Song, W. Shang, P. Tao, T. Deng, Hydrogel-stabilized supercooled salt hydrates for seasonal storage and controlled release of solar-thermal energy. *J. Mater. Chem. A* **12**, 31982–31992 (2024).

19. G. Wang, C. Xu, G. Englmair, W. Kong, J. Fan, S. Furbo, G. Wei, Experimental and numerical study of a latent heat storage using sodium acetate trihydrate for short and long term applications. *J. Energy Storage* **47**, 103588 (2022).

20. P. Tan, P. Lindberg, K. Eichler, P. Löveryd, P. Johansson, A. S. Kalagasidis, Effect of phase separation and supercooling on the storage capacity in a commercial latent heat thermal energy storage: Experimental cycling of a salt hydrate PCM. *J. Energy Storage* **29**, 101266 (2020).

21. B. Sandnes, J. Rekstad, Supercooling salt hydrates: Stored enthalpy as a function of temperature. *Sol. Energy* **80**, 616–625 (2006).

22. M. Goswami, N. Kumar, Y. Li, J. Hirschey, T. J. LaClair, D. O. Akamo, S. Sultan, O. Rios, K. R. Gluesenkamp, S. Graham, Understanding supercooling mechanism in sodium sulfate decahydrate phase-change material. *J. Appl. Phys.* **129**, 245109 (2021).

23. L. Piquard, E. Gagnière, G. Largiller, D. Mangin, F. Bentivoglio, Xylitol used as phase change material: Nucleation mechanisms of the supercooling rupture by stirring. *J. Energy Storage* **48**, 103922 (2022).

24. G. Diarce, A. Rojo, L. Quant, L. Bouzas, A. García-Romero, Thermal endurance of xylitol as a phase change material for thermal energy storage applications. *J. Energy Storage* **55**, 105717 (2022).

25. S. Hirano, H. Takeuchi, Characteristic of reheating operation in supercooled thermal energy storage unit for hot water supply or space heating. *Energy Procedia* **57**, 3140–3149 (2014).

26. M. Yuan, D. Cao, C. Liu, C. Xu, Z. Liao, Temperature-dependent growth kinetic and crystallization behavior of the supercooled erythritol as a phase change material in long-term heat storage. *Chem. Eng. J.* **469**, 143743 (2023).

27. X.-F. Shao, S. Yang, J.-C. Lin, H.-R. Teng, L.-W. Fan, J. N. Chiu, Y.-P. Yuan, V. Martin, Polyvinylpyrrolidone (PVP)-enabled significant suppression of supercooling of erythritol for medium-temperature thermal energy storage. *J. Energy Storage* **46**, 103915 (2022).

28. Y. Wang, S. Li, T. Zhang, D. Zhang, H. Ji, Supercooling suppression and thermal behavior improvement of erythritol as phase change material for thermal energy storage. *Sol. Energy Mater. Sol. Cells* **171**, 60–71 (2017).

29. K. Turunen, V. Mikkola, T. Laukkanen, A. Seppälä, Long-term thermal energy storage prototype of cold-crystallizing erythritol-polyelectrolyte. *Appl. Energy* **332**, 120530 (2023).

30. S. Yang, H. Y. Shi, J. Liu, Y. Y. Lai, Ö. Bayer, L. W. Fan, Supercooled erythritol for high-performance seasonal thermal energy storage. *Nat. Commun.* **15**, 4948 (2024).

31. H. Wang, Z. Rao, L. Li, S. Liao, Phase change characteristics and supercooling suppression of d-mannitol enhanced by ZnO nanoparticles. *J. Energy Storage* **82**, 110627 (2024).

32. X. Hou, Y. Xing, Z. Xu, Y. Du, Y. Gao, J. Yin, S. Wang, Design and experimental analysis of a cooling system with erythritol/xylitol PCM thermal energy storage. *J. Energy Storage* **87**, 111452 (2024).

33. M. R. Yazdani, J. Etula, J. B. Zimmerman, A. Seppälä, Ionic cross-linked polyvinyl alcohol tunes vitrification and cold-crystallization of sugar alcohol for long-term thermal energy storage. *Green Chem.* **22**, 5447–5462 (2020).

34. X. Li, J. Zhang, Y. Liu, Y. Xu, K. Cui, Z. Yao, B. Fu, C. Song, W. Shang, P. Tao, T. Deng, Supercooled sugar alcohols stabilized by alkali hydroxides for long-term room-temperature phase change solar-thermal energy storage. *Chem. Eng. J.* **452**, 139328 (2023).

35. P. G. Debenedetti, F. H. Stillinger, Supercooled liquids and the glass transition. *Nature* **410**, 259–267 (2001).

36. Y. Kashiwakura, T. Sogabe, Y. Hiyama, N. Arakawa, T. Fujii, T. Tochio, K. Kawai, Prediction and control of glass transition temperature for hydrogenated starch hydrolysates and its impact on the texture modification of gummy. *Food Hydrocoll.* **126**, 107467 (2022).

37. J. McFetridge, T. Rades, M. Lim, Influence of hydrogenated starch hydrolysates on the glass transition and crystallisation of sugar alcohols. *Food Res. Int.* **37**, 409–415 (2004).

38. M. Nakanishi, R. Nozaki, Systematic study of the glass transition in polyhydric alcohols. *Phys. Rev. E Stat. Nonlin. Soft Matter Phys.* **83**, 051503 (2011).

39. C. T. Moynihan, A. J. Easteal, J. Wilder, J. Tucker, Dependence of the glass transition temperature on heating and cooling rate. *J. Phys. Chem.* **78**, 2673–2677 (1974).

40. D. Simatos, G. Blond, G. Roudaut, D. Champion, J. Perez, A. L. Faivre, Influence of heating and cooling rates on the glass transition temperature and the fragility parameter of sorbitol and fructose as measured by DSC. *J. Thermal Anal.* **47**, 1419–1436 (1996).

41. C. Gao, H. M. Ma, Enthalpy relaxation in D-sorbitol glass. *J. Therm. Anal. Calorim.* **120**, 1905–1912 (2015).

42. V. Mathot, M. Pyda, T. Pijpers, G. Vanden Poel, E. van de Kerkhof, S. van Herwaarden, F. van Herwaarden, A. Leenaers, The Flash DSC 1, a power compensation twin-type, chip-based fast scanning calorimeter (FSC): First findings on polymers. *Thermochim. Acta* **522**, 36–45 (2011).

43. J. E. K. Schawe, J. F. Löffler, Existence of multiple critical cooling rates which generate different types of monolithic metallic glass. *Nat. Commun.* **10**, 1337 (2019).

44. C. S. Ray, S. T. Reis, R. K. Brow, W. Höland, V. Rheinberger, A new DTA method for measuring critical cooling rate for glass formation. *J. Non Cryst. Solids* **351**, 1350–1358 (2005).

45. Y. Guo, S. R. Byrn, G. Zografi, Physical characteristics and chemical degradation of amorphous quinapril hydrochloride. *J. Pharm. Sci.* **89**, 128–143 (2000).

46. Y. Aso, S. Yoshioka, S. Kojima, Explanation of the crystallization rate of amorphous nifedipine and phenobarbital from their molecular mobility as measured by ^{13}C nuclear magnetic resonance relaxation time and the relaxation time obtained from the heating rate dependence of the glass transition temperature. *J. Pharm. Sci.* **90**, 798–806 (2001).

47. I. M. Hodge, Strong and fragile liquids—A brief critique. *J. Non Cryst. Solids* **202**, 164–172 (1996).

48. R. Böhmer, K. L. Ngai, C. A. Angel, D. J. Plazek, Nonexponential relaxations in strong and fragile glass formers. *J. Chem. Phys.* **99**, 4201–4209 (1993).

49. X. Jin, W. Limin, Enthalpy relaxation studies of memory effect in various glass formers in the vicinity of glass transition. *Acta Phys. Sin.* **66**, 176406 (2017).

50. G. P. Johari, M. Goldstein, Viscous liquids and the glass transition. II. Secondary relaxations in glasses of rigid molecules. *J. Chem. Phys.* **53**, 2372–2388 (1970).

51. M. Gao, J. H. Perepezko, Merging of relaxations and step-like increase of accompanying supercooled liquid region in metallic glasses via ultrafast nanocalorimetry. *NPG Asia Mater.* **14**, 60 (2022).

52. C. A. Angell, Formation of glasses from liquids and biopolymers. *Science* **267**, 1924–1935 (1995).

53. W. H. Wang, Dynamic relaxations and relaxation-property relationships in metallic glasses. *Prog. Mater. Sci.* **106**, 100561 (2019).

54. M. K. Haque, K. Kawai, T. Suzuki, Glass transition and enthalpy relaxation of amorphous lactose glass. *Carbohydr. Res.* **341**, 1884–1889 (2006).

55. D. Tonchev, S. O. Kasap, Effect of aging on glass transformation measurements by temperature modulated DSC. *Mater. Sci. Eng. A* **328**, 62–66 (2002).

56. J. Y. Raty, W. Zhang, J. Luckas, C. Chen, R. Mazzarello, C. Bichara, M. Wuttig, Aging mechanisms in amorphous phase-change materials. *Nat. Commun.* **6**, 7467 (2015).

57. Heat Roadmap Europe, Heat Roadmap Europe (Aalborg University); http://heatroadmap.eu (accessed 4 September 2026).

58. H. Tokoro, M. Yoshikiyo, K. Imoto, A. Nama, T. Nasu, K. Nakagawa, N. Ozaki, F. Hakoe, K. Tanaka, K. Chiba, External stimulation-controllable heat-storage ceramics. *Nat. Commun.* **6**, 7037 (2015).

59. Y. Nakamura, Y. Sakai, M. Azuma, S. Ohkoshi, Long-term heat-storage ceramics absorbing thermal energy from hot water. *Sci. Adv.* **6**, eaaz5264 (2020).

60. P. Dang, J. Hu, Y. Xian, C. Li, Y. Zhou, X. Ding, J. Sun, D. Xue, Elastocaloric thermal battery: Ultrahigh heat-storage capacity based on generative learning-designed phase-change alloys. *Adv. Mater.* **37**, 2412198 (2025).

61. C. A. Angell, K. L. Ngai, G. B. McKenna, P. F. McMillan, S. W. Martin, Relaxation in glass forming liquids and amorphous solids. *J. Appl. Phys.* **88**, 3113–3157 (2000).

62. D. J. Salley, Studies in cellulose deterioration. I. The autoxidation of mannitol. *J. Phys. Chem.* **38**, 449–463 (1934).

63. B. Tong, R.-B. Liu, C.-G. Meng, F.-Y. Yu, S.-H. Ji, Z.-C. Tan, Heat capacities and nonisothermal thermal decomposition reaction kinetics of $_D$-mannitol. *J. Chem. Eng. Data* **55**, 119–124 (2010).

64. A. Mojiri, N. Grbac, B. Bourke, G. Rosengarten, D-mannitol for medium-temperature thermal energy storage. *Sol. Energy Mater. Sol. Cells* **176**, 150–156 (2018).

65. A. Yamaguchi, N. Hiyoshi, O. Sato, M. Shirai, Sorbitol dehydration in high-temperature liquid water. *Green Chem.* **13**, 873–881 (2011).
